# [3 + 2]-Cycloaddition reaction of sydnones with alkynes

**DOI:** 10.3762/bjoc.14.113

**Published:** 2018-06-05

**Authors:** Veronika Hladíková, Jiří Váňa, Jiří Hanusek

**Affiliations:** 1 Institute of Organic Chemistry and Technology, University of Pardubice, Studentská 573, 532 10 Pardubice, Czech Republic

**Keywords:** alkynes, Cu(I) catalysis, [3 + 2]-cycloaddition, mechanism, regioselectivity, sydnones

## Abstract

This review covers all known examples of [3 + 2]-cycloaddition between sydnones and both terminal as well as internal alkynes/cycloalkynes taken from literature since its discovery by Huisgen in 1962 up to the current date. Except enumeration of synthetic applications it also covers mechanistic studies, catalysis, effects of substituents and reaction conditions influencing reaction rate and regioselectivity.

## Review

### Introduction

Since Huisgen’s discovery of the [3 + 2]-cycloaddition between 3-substituted sydnones and both terminal as well as internal alkynes [1–2] many researchers have tried to utilize this synthetic approach for the synthesis of polysubstituted 1,2-diazoles (pyrazoles, indazoles). However, until 2013 when Taran’s group introduced the regioselective Cu(I)-phenanthroline catalysis [3] this method was of limited value due to the harsh reaction conditions and sometimes also due to low regioselectivity in those cases when a non-symmetrical alkyne was employed as a reactant. Surprisingly, until the fall of 2017, no comprehensive work concerning this important topic was published. This encouraged us to write this review. During its completion a new feature article bridging this gap was published by Taran et al. [4]. In order to avoid duplication our review is therefore focused in more detail on thermal, photochemical as well as metal-catalyzed reactions of sydnones with alkynes and factors that influence the yield and ratio of both possible regioisomers and also the kinetics and mechanism of this cycloaddition reaction.

### Thermal reaction of sydnones with symmetrical alkynes and cycloalkynes

**Scheme 1:**
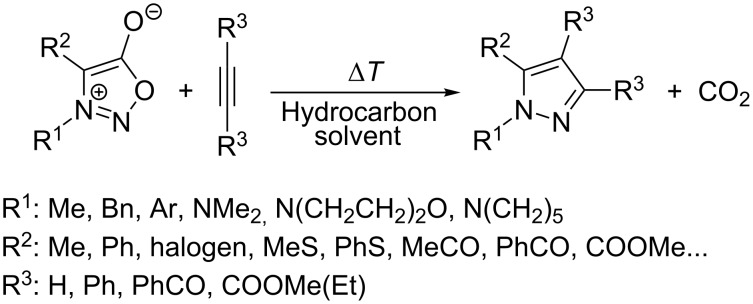
Thermal reaction of sydnones with symmetrical alkynes.

As mentioned above, the thermal reaction of 3-alkyl-, 3-aryl- or even 3-substituted aminosydnones with symmetrical alkynes (Scheme 1) represents a very useful and straightforward method for the synthesis of substituted 1,3,4-tri- or 1,3,4,5-tetrasubstituted pyrazoles [1–2,5–39] or indazoles.

Dimethyl acetylenedicarboxylate (DMAD, R^3^ = COOMe) or its analogues (diethyl; R^3^ = COOEt); di-*tert*-butyl, R^3^ = COO*t*-Bu etc.) act as the most common dipolarophiles because of their high reactivity. Moreover, one or both carboxylate groups in position 3 and 4 in the final pyrazole are easily removable using a hydrolysis/decarboxylation protocol [5,16] thus giving pyrazole-4-carboxylic acids – potent xanthine oxidoreductase inhibitors [40] or even 3,4-unsubstituted pyrazoles [16]. Both pyrazole carboxylic groups can be also modified to hydrazides and oxazole rings [26] or a new condensed pyridazine ring [27]. Less reactive dipolarophiles such as dibenzoylacetylenes (1,4-diphenylbut-2-yn-1,4-diones) [13,17,38–39], diphenylacetylene [1–2,9,13,15,32] or even acetylene itself [1–2] have also been successfully reacted with sydnones. The most typical procedure involves heating both components in boiling hydrocarbon solvent (benzene, toluene or xylene) for several hours (up to 24 h) and the isolated yields are often close to 90% for the ordinary substituents (alkyls, aryls, halogens) of the sydnone. Somewhat lower yields were obtained in ethyleneglycol [5]. The reaction of the parent 1-phenylsydnone with DMAD and its diethyl analogue has also been performed in supercritical carbon dioxide [41] in which 65 and 83% yields of dimethyl (or diethyl) 1-phenylpyrazole-3,4-dicarboxylates were achieved. Only in two cases involving 3-(2,4,6-trisubstituted phenyl)-4-iodosydnones (R^1^: 2-Br-4,6-diMe-Ph, 2,4-diBr-6-Me-Ph; R^2^: I) and DMAD (or its diethyl analogue) did the cycloaddition completely fail [21–22] even after heating for 3 days in boiling xylene. This result was explained by the steric hindrance between the bulky substituents in the 4-position (iodine) and the substituents (Me, Br) in both *ortho*-positions of the adjacent 2,4,6-trisubstituted phenyl ring. All the examples found for [3 + 2]-cycloadditions between sydnones and symmetrical non-cyclic alkynes including conditions used for the synthesis, are presented in Table 1.

**Table 1:** Thermal cycloaddition of sydnones with symmetrical non-cyclic alkynes.

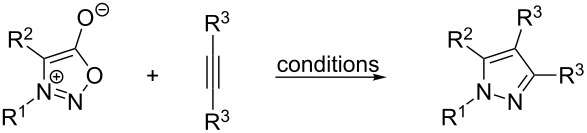						

entry	R^1^	R^2^	R^3^	conditions	yield [%]	ref.

1	Ph	H	H	acetone, 170 °C, 25 h	75	[1–2]
2	Ph	Me	Ph	180 °C, 5 h	96–97	[1–2]
3	Ph	H	Ph	160 °C, 4.5 h	93	[2]
4	Ph	Ph	Ph	190 °C, 9 h	98	[2]
5	Ph	H	COOMe	toluene, 90 °C, 4 h xylene, reflux *p*-xylene, reflux, overnight *p*-xylene, reflux, overnight *p*-xylene, reflux, 4 h	92 92 98 93 93	[1–2] [20] [26] [29] [31]
6	Ph	Me	COOMe	xylene, 120 °C, 1 h	99	[1–2]
7	Bn	H	COOMe	xylene, 120 °C, 5 h	93–98	[1–2]
8	Ph	Cl	COOMe	ethyleneglycol, 120 °C, 1 h xylene, reflux	74 60–80	[5] [6]
9	Ph	Br	COOMe	ethyleneglycol, 120 °C, 1 h xylene, reflux	70 60–80	[5] [6]
10	Me	Cl	COOMe	ethyleneglycol, 120 °C, 1.5 h	12	[5]
11	Me	Br	COOMe	ethyleneglycol, 120 °C, 1.5 h	82	[5]
12	Ph	NO_2_	COOMe	xylene, reflux	60–80	[6]
13	4-Br-Ph	H	COOMe	xylene, reflux *p*-xylene, reflux, 6 h	60–80 92	[6] [35]
14	4-Br-Ph	Br	COOMe	xylene, reflux	60–80	[6]
15	4-Br-Ph	Cl	COOMe	xylene, reflux	60–80	[6]
16	4-Cl-Ph	H	COOMe	xylene, reflux *p*-xylene, reflux, overnight	60–80 98	[6] [26]
17	4-Cl-Ph	Br	COOMe	xylene, reflux	60–80	[6]
18	4-Cl-Ph	Cl	COOMe	xylene, reflux	60–80	[6]
19	4-MeO-Ph	H	COOMe	xylene, reflux xylene, reflux, overnight *p*-xylene, reflux, 4 h	60–80 91 91	[6] [29] [31]
20	4-MeO-Ph	Br	COOMe	xylene, reflux	60–80	[6]
21	4-MeO-Ph	Cl	COOMe	xylene, reflux	60–80	[6]
22	4-Br-3-Cl-Ph	H	COOMe	xylene, reflux	89	[7]
23	4-Br-3-Cl-Ph	Br	COOMe	xylene, reflux	71	[7]
24	4-Br-3-Cl-Ph	Cl	COOMe	xylene, reflux	61	[7]
25	4-NO_2_-Ph	H	COOMe	toluene, 110 °C, 1.75 h *p*-xylene, reflux, overnight	99 98	[8] [26]
26	4-NO_2_-Ph	Ph	COOMe	toluene, 100–105 °C, 16 h	96	[8]
27	2,4-di-NO_2_-Ph	Ph	COOMe	toluene, 100–105 °C, 4 h	97	[8]
28	Ph	MeS	COOMe	toluene, 100 °C, 2 h	96	[8]
29	4-Me_2_N-Ph	MeS	COOMe	mesitylene, 130–135 °C, 0.5 h	92	[8]
30	Ph	PhS	COOMe	xylene, 120–125 °C, 5.75 h	91	[8]
31	Ph	PhS=O	COOMe	mesitylene, 135–140 °C, 26 h	63	[8]
32	Ph	MeC=O	COOMe	xylene, 160 °C, 18 h	62	[8]
33	4-MeO-Ph	MeC=O	COOMe	mesitylene, 160–165 °C, 22 h	95	[8]
34	4-MeO-Ph	CN	COOMe	xylene, 160 °C, 24 h	79	[8]
35	Me_2_N	MeS	COOMe	xylene, 160 °C, 18 h benzene, 80 °C, 16 h	31 19	[9]
36	Me_2_N	MeS	Ph	xylene, 155–160 °C, 93 h	71	[9]
37	Me_2_N	PhS	COOMe	xylene, 155–160 °C, 19 h benzene, 80 °C, 23 h	30 0	[9]
38	Me_2_N	H	COOMe	xylene, 155–160 °C, 3 h benzene, 80 °C, 19 h	9 2	[9]
39	Me_2_N	CN	COOMe	xylene, 155–160 °C, 3 h	0	[9]
40	O(CH_2_CH_2_)_2_N	MeS	COOMe	benzene, 80 °C, 23 h	53	[9]
41	O(CH_2_CH_2_)_2_N	PhS	COOMe	xylene, 155–160 °C, 22 h	70	[9]
42	(CH_2_)_5_N	MeS	COOMe	xylene, 160 °C, 20 h	47	[9]
43	(CH_2_)_5_N	PhS	COOMe	xylene, 150–160 °C, 24 h	27	[9]
44	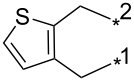		COOMe	benzene, reflux	71	[10]
45	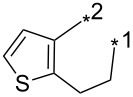		COOMe	benzene, reflux	77	[10]
46	4-MeCO-Ph	H	COOMe	xylene, reflux	56	[11]
47	4-MeCO-Ph	Me	COOMe	xylene, reflux	51	[11]
48	4-MeCO-Ph	Ph	COOMe	xylene, reflux	38	[11]
49	4-(Me(Ph)NSO_2_)-Ph	H	COOMe	xylene, reflux, 2 h	75	[12]
50	4-(Et(Ph)NSO_2_)-Ph	H	COOMe	xylene, reflux, 2 h	75	[12]
51	4-(O(CH_2_CH_2_)_2_NSO_2_)-Ph	H	COOMe	xylene, reflux, 2 h	78	[12]
52	4-((CH_2_)_5_NSO_2_)-Ph	H	COOMe	xylene, reflux, 2 h	76	[12]
53	4-((CH_2_)_4_NSO_2_)-Ph	H	COOMe	xylene, reflux, 2 h	75	[12]
54	4-(Et_2_NSO_2_)-Ph	H	COOMe	xylene, reflux, 2 h	75	[12]
55	4-(O(CH_2_CH_2_)_2_NSO_2_)-Ph	Br	COOMe	xylene, reflux, 2 h	66	[12]
56	4-((CH_2_)_5_NSO_2_)-Ph	Br	COOMe	xylene, reflux, 2 h	70	[12]
57	CH_2_CH_2_CH_2_		Ph	xylene, reflux, 48 h	45	[13]
58	CH_2_CH_2_CH_2_		COOMe	xylene, reflux, 8 h	80	[13]
59	CH_2_CH_2_CH_2_		PhCO	xylene, reflux, 8 h	92	[13]
60	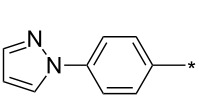	Me	COOMe	xylene, 120 °C	–	[14]
61	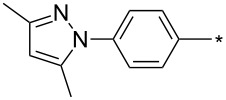	Me	COOMe	xylene, 120 °C	–	[14]
62	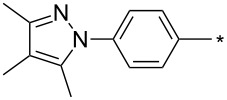	Me	COOMe	xylene, 120 °C	–	[14]
63	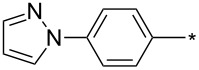	Ph	COOMe	xylene, 120 °C	–	[14]
64	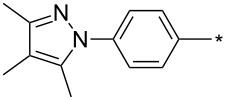	Ph	COOMe	xylene, 120 °C	–	[14]
65	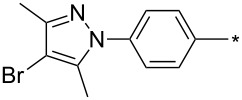	Br	COOMe	xylene, 120 °C	–	[14]
66	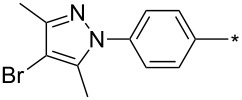	Ph	COOMe	xylene, 120 °C	–	[14]
67	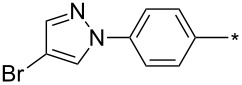	Ph	COOMe	xylene, 120 °C	–	[14]
67	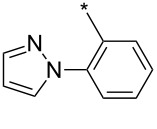	Me	COOMe	xylene, 120 °C	–	[14]
68	CH_2_CH_2_CH_2_CH_2_		Ph	*p*-xylene, reflux, 24 h,	91	[15]
69	Me	H	Ph	160 °C, 7 d	16	[15]
70	Me	Ph	COOMe	*p*-xylene, reflux, overnight	77	[16]
71	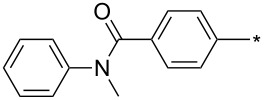	H	COOMe	xylene, reflux	52	[17]
72	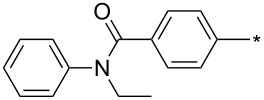	H	COOMe	xylene, reflux	60	[17]
73	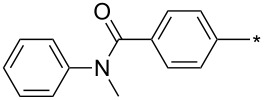	H	PhCO	xylene, reflux	50	[17]
74	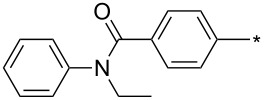	H	PhCO	xylene, reflux	53	[17]
75	Ph	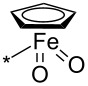	COOMe	toluene, reflux	67	[18]
76	Ph	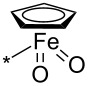	COO*t*-Bu	toluene, reflux	48	[18]
77	Me	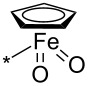	COOMe	toluene, reflux	54	[18]
78	Ph	H	Bu_3_Sn	xylene, reflux, 16 h	98	[19]
79	Me	I	COOMe	toluene + DMSO, reflux, 6 h	84	[20]
80	CH_2_CH_2_CN	I	COOMe	toluene, reflux, 6 h	95	[20]
81	Ph	I	COOMe	toluene, reflux, 6 h	80	[20]
82	2-Me-Ph	I	COOMe	toluene, reflux, 6 h	88	[20]
83	2-Et-Ph	I	COOMe	toluene, reflux, 6 h	83	[20]
84	2-MeO-Ph	I	COOMe	toluene, reflux, 6 h	83	[20]
85	3-MeO-Ph	I	COOMe	toluene, reflux, 6 h	84	[20]
86	4-MeO-Ph	I	COOMe	toluene, reflux, 6 h	90	[20]
87	2-Me-Ph	Cl	COOMe	toluene, reflux, 6 h	–	[20]
88	2-Et-Ph	Br	COOMe	toluene, reflux, 6 h	92	[20]
89	2,4-diMe-Ph	I	COOMe	toluene, reflux	87	[21]
90	2,4-diMe-6-Br-Ph	H	COOMe	toluene, reflux	82	[21]
91	2,4-diBr-6-Cl-Ph	H	COOMe	toluene, reflux	90	[21]
92	2-Br-4,6-diMe-Ph	I	COOMe	toluene, reflux	0	[21]
93	4-Br-2-Me-Ph	H	COOMe	xylene, reflux, 8 h	83	[22]
94	4-Br-2-Me-Ph	Cl	COOMe	xylene, reflux, 8 h	81	[22]
96	4-Br-2-Me-Ph	Br	COOMe	xylene, reflux, 8 h	88	[22]
97	4-Br-2-Me-Ph	I	COOMe	xylene, reflux, 8 h	79	[22]
98	4,6-Br_2_-2-Me-Ph	H	COOMe	xylene, reflux, 8 h	92	[22]
99	4-Br-2-Me-Ph	H	COOEt	xylene, reflux, 8 h	82	[22]
100	2,4-Br_2_-6-Me-Ph	I	COOMe(Et)	xylene, reflux, 3 d	0	[22]
101	2-Cl-Ph	I	COOMe	xylene, reflux, 8 h	78	[23]
102	2-Cl-4-Br-Ph	I	COOMe	xylene, reflux, 8 h	87	[23]
103	2-Cl-4-Br-Ph	H	COOMe	xylene, reflux, 8 h	91	[23]
104	4-Br-2-Et-Ph	H	COOMe	toluene, reflux, 10 h	82	[24]
105	4-Br-2-Me-Ph	I	COOMe	toluene, reflux, 10 h	90	[24]
106	2,5-diMe-Ph	I	COOMe	toluene, reflux, 8 h	85	[25]
107	5-Cl-2-Me-Ph	I	COOMe	toluene, reflux, 8 h	82	[25]
108	2,5-diMe-Ph	Br	COOMe	toluene, reflux, 8 h	83	[25]
109	5-Cl-2-Me-Ph	Br	COOMe	toluene, reflux, 8 h	87	[25]
110	2,4-diMe-Ph	Br	COOMe	toluene, reflux, 8 h	81	[25]
111	2,4-diMe-Ph	Cl	COOMe	toluene, reflux, 8 h	80	[25]
112	2,5-diMe-Ph	H	COOMe	toluene, reflux, 8 h	80	[25]
113	5-Cl-2-Me-Ph	H	COOMe	toluene, reflux, 8 h	80	[25]
114	2,4-diMe-Ph	H	COOMe	toluene, reflux, 8 h	80	[25]
115	4-EtOOC-Ph	H	COOMe	*p*-xylene, reflux, overnight	98	[26]
116	4-Me-Ph	H	COOMe	*p*-xylene, reflux, overnight	98	[26]
117	4-EtO-Ph	H	COOMe	*p*-xylene, reflux, overnight xylene, 120 °C, 1 h	98 94	[26] [27]
118	3-Cl-4-Me-Ph	H	COOMe	xylene, 120 °C, 1 h	99	[27]
119	3-NO_2_-4-Me-Ph	H	COOMe	xylene, 120 °C, 1 h	96	[27]
120	2,3-diMe-Ph	H	COOMe	toluene, reflux, 10 h	89	[28]
121	2,3-diMe-Ph	Cl	COOMe	toluene, reflux, 10 h	76	[28]
122	2,3-diMe-Ph	Br	COOMe	toluene, reflux, 10 h	75	[28]
123	2,3-diMe-Ph	I	COOMe	toluene, reflux, 10 h	77	[28]
124	2,3-diMe-Ph	H	COOCH_2_CF_3_	toluene, reflux, 12 h	83	[28]
125	Ph	Ph	COOMe	toluene, reflux, 16 h	99	[30]
126	Ph	4-NO_2_-Ph	COOMe	toluene, reflux, 16 h	87	[30]
127	Ph	4-OCH_3_-Ph	COOMe	toluene, reflux, 16 h	82	[30]
128	Ph	CF_3_	Ph	*o*-dichlorobenzene, 24 h, 180 °C	53	[32]
129	Bn	CF_3_	COOMe	*o*-dichlorobenzene, 20 h, 120 °C (180 °C)	54^a^ (51)^a^	[33]
130	Ph	CH_2_F → CH_2_OH	COOMe	*o*-dichlorobenzene, 24 h, 100 °C	57	[33]
131	Ph	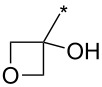	COOMe	*o*-dichlorobenzene, 5 min, 180 °C (μ-wave)	92	[34]
132	4-MeO-Ph	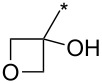	COOMe	*o*-dichlorobenzene, 20 min, 180 °C (μ-wave)	60	[34]
133	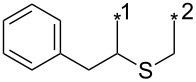		COOMe	xylene, reflux, 3 h	70	[36]
134	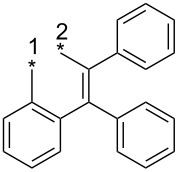		COOMe	toluene, 115 °C, overnight	81	[52]
135	2-MeO-Ph	H, Br, Cl	COOMe	xylene, reflux	n.d.	[37]
136	2-NO_2_-Ph	H, Br, Cl	COOMe	xylene, reflux	n.d.	[37]
137	3-NO_2_-Ph	H, Br, Cl	COOMe	xylene, reflux	n.d.	[37]
138	2-Cl-Ph	H, Br, Cl	COOMe	xylene, reflux	n.d.	[37]
139	3-Cl-Ph	H, Br, Cl	COOMe	xylene, reflux	n.d.	[37]
140	Ph	Ph	PhCO	toluene, heating, 92 h	69	[38]
141	Ph	H	PhCO	PEG, 115 °C, 3 min, (μ-wave)	50	[39]
142	4-Cl-Ph	H	PhCO	PEG, 115 °C, 3 min, (μ-wave)	51	[39]
143	4-Me-Ph	H	PhCO	PEG, 115 °C, 3 min, (μ-wave)	54	[39]
144	Ph	H	4-MeOPhCO	PEG, 115 °C, 3 min, (μ-wave)	48	[39]
145	4-Cl-Ph	H	4-MeOPhCO	PEG, 115 °C, 3 min, (μ-wave)	48	[39]
146	4-Me-Ph	H	4-MeOPhCO	PEG, 115 °C, 3 min, (μ-wave)	49	[39]

^a^Mixture of dimethyl 1-benzyl-5-trifluoromethyl-1*H*-pyrazole-3,4-dicarboxylate and dimethyl 1-benzyl-3-trifluoromethyl-1*H*-pyrazole-4,5-dicarboxylate in the ratio 96:4 (at 120 °C) or 63:37 (at 180 °C).

**Scheme 2:**
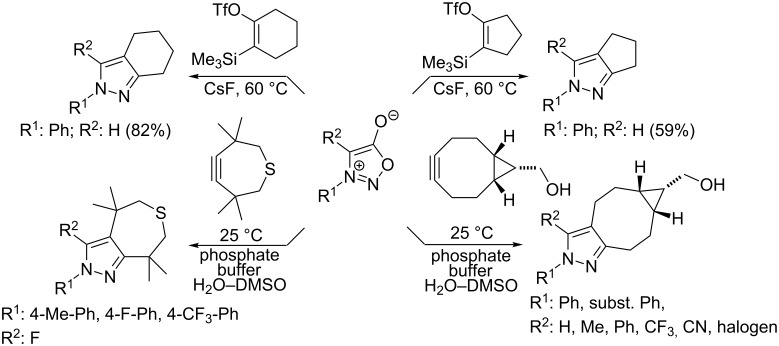
Reaction of sydnones with strained cycloalkynes.

Extraordinarily good dipolarophiles – e.g., cycloalkynes – containing a very reactive “bent” triple bond such as in bicyclo[6.1.0]non-4-yne-9-methanol [42–44] or in 3,3,6,6-tetramethylthiacyclohept-4-yne [44] were recently suggested as highly reactive partners for bio-orthogonal ligation reactions [45–46]. It is also possible to generate highly unstable cyclopentyne or cyclohexyne in situ from the corresponding 2-trimethylsilylcycloalken-1-yl triflates [47] and trap them by reaction with sydnones (Scheme 2).

**Scheme 3:**
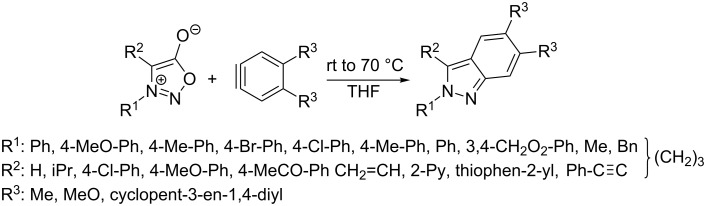
Reaction of sydnones with didehydrobenzenes.

These strain-promoted reactions proceed quickly under very mild conditions (at room temperature, in aqueous phosphate buffer with solubilizing DMSO). In a similar manner, very reactive benzynes (didehydrobenzenes) generated either from 2-aminobenzoic acid [48], from symmetrically substituted 2-trimethylsilylphenyl triflates [49–52] or from 2-(trimethylsilyl)phenyl trimethylsilyl ethers [53] react with sydnones in MeCN or THF giving 2-substituted 2*H*-indazoles in good to excellent yields (40–99%) at room temperature (Scheme 3).

**Scheme 4:**
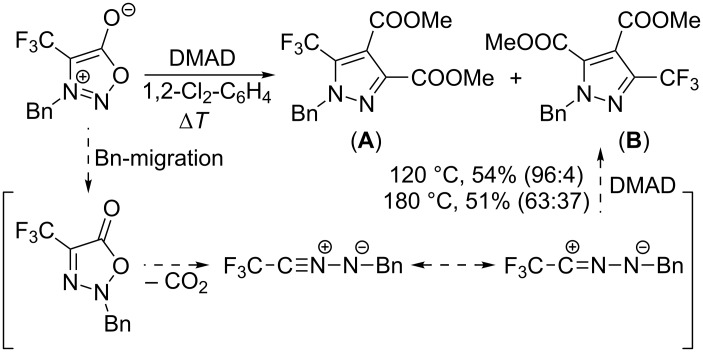
Formation of isomeric pyrazole dicarboxylates.

It was also observed, that formation of isomeric pyrazole-4,5-dicarboxylates (**B**) can sometimes accompany the production of pyrazole-3,4-dicarboxylates (**A**) under thermal conditions [33] although their formation is not photoinduced (cf. next chapter) because the reaction also takes place in the absence of light. Depending on the temperature, a new reaction pathway involving benzylic group migration, CO_2_ extrusion and final cycloaddition was proposed (Scheme 4).

### Kinetics and mechanism of thermal cycloaddition

**Scheme 5:**
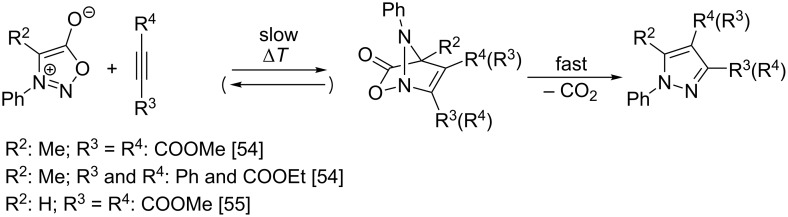
Mechanism of thermal cycloaddition between sydnones and alkynes.

The kinetics and reaction mechanism of the thermal cycloaddition between 4-methyl-3-phenylsydnone and DMAD was first studied by Huisgen and Gotthardt [54] in *p*-cymene at 90–110 °C. They found the cycloaddition to be overall second order and its activation entropy Δ*S*^≠^ = −130 J·mol^−1^·K^−1^ showed association character of the rate-limiting step with a relatively tight transition state. Moreover, for the cycloaddition of the structurally similar ethyl phenylpropiolate in various solvents only a small decrease of the bimolecular rate constant with increasing solvent polarity (in terms of relative permittivity) was observed excluding a transition state having a polarized character. Finally, substitution effects in the 3-(4-substituted phenyl) group of sydnones were studied and a relatively low Hammett reaction constant ρ ≈ +0.8 was estimated from four derivatives (MeO, Me, H and Cl). An even smaller dependence of the rate constants on the solvent polarity and substituent effect sensitivity (ρ ≈ +0.3 to +0.4) was described [55] for reactions of 3-(4-substituted phenyl)sydnones with more reactive DMAD while the activation entropy (Δ*S*^≠^ = −106 to −121 J·mol^−1^·K^−1^) remained similar. The reaction mechanism (Scheme 5) consistent with these kinetic measurements involves rate-limiting formation of a bicyclic intermediate via a concerted [3 + 2]-cycloaddition followed by its very fast decomposition (extrusion of CO_2_) via a retro-Diels–Alder [4 + 2]-cycloaddition. The almost spontaneous extrusion of CO_2_ is caused by an energetically favorable aromatization occurring in this step leading to the formation of the stable pyrazole ring. Both reaction steps are also compatible with Woodward–Hoffmann rules, taking into account orbital symmetry considerations [56].

Three types of [3 + 2]-cycloadditions (labelled I–III) are known from the literature [57] each differing in the frontier molecular orbital energies between the dipole and dipolarophile. While for type I (HOMO-controlled) combining a high-lying dipole HOMO with a dipolarophile LUMO the reaction is accelerated by electron-donating substituents on the dipole and electron-withdrawing substituents on the dipolarophile (both lowering the HOMO–LUMO energy gap), for type III (LUMO-controlled) combining a low-lying dipole LUMO and a dipolarophile HOMO where substituent effects are completely opposite. For type II cycloadditions in which two-way interactions between the dipole HOMO and the dipolarophile LUMO or the dipole LUMO and the dipolarophile HOMO are possible – due to similar energy gaps – both electron-rich as well as electron-poor dipolarophiles/dipoles react more quickly than parent (unsubstituted) ones. Using semi-empirical quantum calculations (CNDO/2), Houk et al. [58] calculated average HOMO/LUMO energies for azomethine-imines (ε_HOMO_ = −8.6 eV and ε_LUMO_ = 0.3 eV) and predicted that the ε_LUMO_ for structurally related sydnones containing an electron-withdrawing –COO– motif should be even much lower suggesting a LUMO-controlled reaction (type III). Such a prediction seems to be correct for reaction of 4-(substituted phenyl)sydnones with DMAD for which positive Hammett ρ-values were observed [54–55]. On the other hand Huisgen and Gotthardt [54] measured bimolecular rate constants for the above-mentioned reaction of 4-methyl-3-phenylsydnone and various acetylenes in *p*-cymene at 140 °C (Table 2) and found a reactivity sequence corresponding rather to type II or even type I cycloadditions.

**Table 2:** Bimolecular rate constants (*k*, L·mol^–1^·s^–1^) measured for the reaction of 4-methyl-3-phenylsydnone and various acetylenes in *p*-cymene at 140 °C [54].

dipolarophile (disubstituted alkyne)	10^5^*k* (L·mol^−1^·s^−1^)	dipolarophile (monosubstituted alkyne)	10^5^*k* (L·mol^−1^·s^−1^)

MeOOC–C≡C–COOMe	2 580	H–C≡C–COOMe	823
Ph–C≡C–COPh	135	H–C≡C–CH(OPr)_2_	39
Ph–C≡C–COOEt	99	H–C≡C–Ph	18
Ph–C≡C–Ph	3.0^a^	H–C≡C–(CH_2_)_11_CH_3_	6.0
Ph–C≡C–Me	1.9		

^a^In decaline.

The most reactive were electron-poor alkynes (acetylene(di)carboxylates, benzoyl phenylacetylene) while electron-rich alkynes (tetradec-1-yne, 1-phenylpropyne) were much less reactive. Unfortunately, the reaction rate constant was not measured for the reaction with acetylene itself. However, on the basis of the published [1–2] synthetic protocol (acetone, 170 °C, 25 h) it appears that this cycloaddition is very slow and requires a higher temperature.

Recently [42–44], a kinetic investigation was performed for the cycloaddition of various sydnones with strained cycloalkynes such as bicyclo[6.1.0]non-4-yne-9-methanol (BCN) or 3,3,6,6-tetramethylthiacyclohept-4-yne (TMTH). It was found that the reaction of BCN with 3-(4-substituted phenyl)sydnones roughly obeys a Hammett correlation with ρ ≈ +1.35 ± 0.25 [43] thus indicating a type III mechanism. However, the effect of substituent in position 4- of 3-phenylsydnone is ambiguous. While all halogens substantially accelerate the reaction rate (F > Cl > Br > I) other substituents cause up to tenfold deceleration (H > Me > CF_3_ > CN) regardless of their polar effects [43–44]. Steric factors cannot explain the influence of 4-substituent because 4-phenylsydnone reacts equally as unsubstituted one. The most reactive 4-fluoro-3-phenylsydnones [44] were found to react with BCN and TMTH in two kinetically independent reaction steps corresponding to fast formation of the addition intermediate and its slow decomposition to pyrazole and CO_2_. Such ambiguous substitution effects are therefore worthy of further investigations.

### Photochemical reaction of sydnones with symmetrical alkynes

**Scheme 6:**
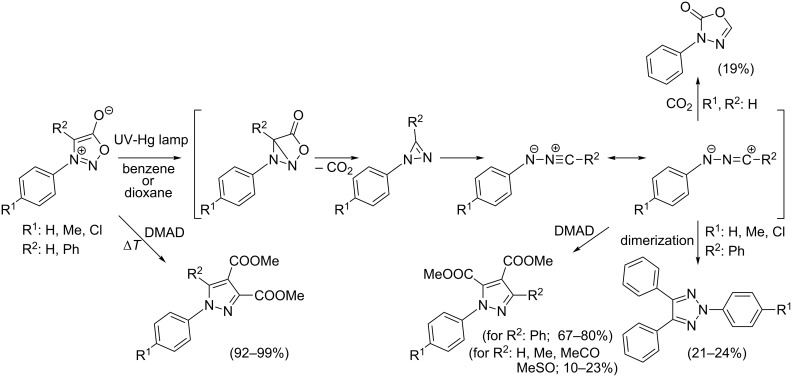
Mechanism of photochemical reaction of sydnones with symmetrical alkynes.

In 1966 Krauch et al. [59] dealt with irradiation (using a high-pressure Hg lamp) of benzene or dioxane solutions of 3-phenylsydnone and proposed formation of *N*-phenylnitrilimine as the main reaction product via an internal ring closure, extrusion of CO_2_ and ring opening (Scheme 6). This very reactive 1,3-dipole was trapped by reaction with external (^14^C-labelled) CO_2_ to give 3-phenyl-1,3,4-oxadiazol-2(3*H*)-one (Scheme 6). A similar experiment was performed by Ohta et al. [60] five years later who irradiated single 3,4-diphenylsydnones and obtained the corresponding 2,4,5-triphenyl-1,2,3-triazoles in 21–24% yields (first misinterpreted as 1,3-diphenyldiazirine [61]). In the same year Angadiyavar and George [62], Gotthardt and Reiter [63–64] and Märky, Hansen and Schmid [65] found that irradiation of a mixture of 3-phenylsydnone or 3,4-diphenylsydnones together with DMAD gave different isomeric [3 + 2]-cycloadducts (pyrazole-4,5-dicarboxylates) than what were obtained under thermal conditions and proved the reaction pathway to proceed via the corresponding *N*-phenylnitrilimine.

The yields (Table 3) are generally lower than those of reactions performed under thermal conditions – most probably due to the lower stability of the key intermediate – *N*-phenylnitrilimine – which can undergo dimerization or reverse trapping of evolved CO_2_. Yields are always much better for 3,4-diarylsydnones for which the corresponding *N*-phenylnitrilimine is resonance-stabilized. The yields also depend on the photoreactor construction [64]. For example 1,3-diphenylsydnone reacts with DMAD in a batch reactor (Rayonet) under 300 nm irradiation to give only 29% of dimethyl 1,3-diphenylpyrazole-4,5-dicarboxylate while in a wetted-wall photo reactor (Normag) the yield is increased up to 84% (at 17 °C in DCM).

**Table 3:** Photochemical cycloaddition of *N*-phenylsydnones with DMAD.

entry	R^1^	R^2^	conditions	yield [%]	ref.

1	Ph	H	CH_2_Cl_2_, 50 h, light (300 nm)	10	[63–64]
2	Ph	Me	CH_2_Cl_2_, 19 h, light (300 nm)	23	[63–64]
3	Ph	Ph	benzene, 2 h, CH_2_Cl_2_, light (300 nm), batch CH_2_Cl_2_, 29.5 h, light (300 nm) wetted-wall photoreactor dioxane, Hg lamp	67 29 29 84 ca. 80	[62] [63] [64] [64] [65]
4	Ph	4-Me-Ph	CH_2_Cl_2_, Hg lamp	ca. 80	[65]
5	Ph	MeS	benzene, 25 h, Hg lamp	45	[64]
6	Ph	MeSO	CH_2_Cl_2_, 110 h, Hg lamp	12	[64]
7	Ph	MeCO	benzene, 41 h, Hg lamp	17	[64]

### Thermal reaction of sydnones with terminal alkynes

As early as in his first work [1] dealing with sydnone–alkyne cycloaddition Huisgen et al. found that some non-symmetrical alkynes (oct-1-yne, phenylacetylene and especially methyl propiolate) gave mixture of both pyrazole regioisomers. The following Table 4 summarizes all known examples [1–2,8,20,24,32–34,36,66–93] where the ratio of both possible regioisomers or at least chemical yield of the major regioisomer was given.

**Table 4:** Thermal cycloaddition of sydnones with terminal alkynes.

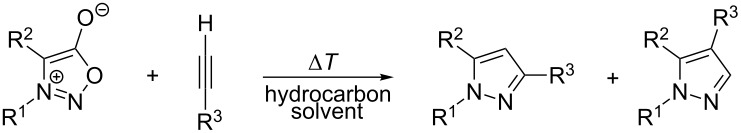							

entry	R^1^	R^2^	R^3^	conditions	ratio 1,3:1,4	yield [%]^a^	ref.

1	Ph	Me	*n*-Hex	xylene, 140 °C, 30 h	n.d.	78	[1]
2	Ph	H	*n*-Hex	toluene, 111 °C, 52 h xylene, 160 °C, 24 h	n.d. 90:10	72 65	[2] [91]
3	Ph	H	Ph	chlorobenzene, 120 °C, 20 h xylene, 140 °C, 16 h *o*-DCB, μ-wave, 200 °C, 2 h *o*-DCB, 140 °C, 24 h	n.d. >95:5 91:9 91:9	79/<2 35 66 62	[1–2] [82] [84] [92]
4	Ph	Me	Ph	140 °C, 12 h 142 °C, 7 h	~80:20 ~89:11	64/15 73/9	[1] [2]
5	Bn	H	Ph	xylene, 135–140 °C, 20 h	100:0	69–74	[1–2]
6	Ph	H	COOMe	xylene, 100 °C, 48 h sc-CO_2_, 60–160 °C, 7.6 MPa	76:24 85:15–76:24	70/22 –	[1–2] [93]
7	Ph	Me	COOMe	140 °C, 4 h xylene, reflux, 1 h	n.d. 65:35	61/10 55/29	[1] [2]
8	Ph	H	CH(OPr)_2_	xylene, 135–140 °C, 3 h	n.d.	28/58	[2]
9	Ph	Me	CH(OPr)_2_	xylene, 135–140 °C, 15 h	n.d.	77	[1]
10	Bn	H	CH(OPr)_2_	xylene, 135–140 °C, 15 h	n.d	78	[2]
11	Ph	H	CH_2_OH	reflux, 24 h	100:0	66–72	[1–2]
12	Ph	H	CN	chlorobenzene, 110 °C, 24 h	100:0	50	[66]
13	NMe_2_	H	Ph	tetraline, reflux, 5 h	n.d.	60/–	[67]
14	NMe_2_	H	4-Cl-Ph	tetraline, reflux, 5 h	n.d.	23/–	[67]
15	NMe_2_	H	4-Me-Ph	tetraline, reflux, 5 h	n.d.	32/–	[67]
16	NMe_2_	H	*n*-Hex	tetraline, reflux, 5 h	n.d.	50/–	[67]
17	O(CH_2_CH_2_)_2_N	H	Ph	tetraline, reflux, 5 h	n.d.	22/–	[67]
18	(CH_2_)_5_N	H	4-Cl-Ph	tetraline, reflux, 5 h	n.d.	24/1	[67]
19	NMe_2_	Me	Ph	tetraline, reflux, 5 h	n.d.	81/10	[67]
20	NMe_2_	Me	4-Cl-Ph	tetraline, reflux, 5 h	n.d.	30/4	[67]
21	NMe_2_	O(CH_2_CH_2_)_2_NCH_2_	Ph	tetraline, reflux, 5 h	n.d.	34/2	[67]
22	NMe_2_	O(CH_2_CH_2_)_2_NCH_2_	4-Cl-Ph	tetraline, reflux, 5 h	n.d.	12/2	[67]
23	Ph	MeS	COOMe	toluene, 95–105 °C, 12.5 h	46:54	39/50	[8]
24	Ph	PhS	COOMe	xylene, 140 °C, 35 h	53:47	95	[8]
25	Ph	MeSO	COOMe	mesitylene, 135–140 °C, 19 h	81:19	65/15	[8]
26	Ph	MeCO	COOMe	mesitylene, 155–160 °C, 90 h	60:40	46/37	[8]
27	Ph	Ph	COOMe	xylene, 110–115 °C, 12 h *o*-DCB, reflux, 48 h	50:50 50:50	40/44 97	[8] [80]
28	4-NO_2_-Ph	Ph	COOMe	toluene, 95–105 °C, 16 h	56:44	51/37	[8]
29	2,4-diNO_2_-Ph	Ph	COOMe	toluene, 100–105 °C, 18.5 h	61:39	55/36	[8]
30	4-NO_2_-Ph	H	COOMe	toluene, 95–105 °C, 4 h	86:14	99	[8]
31	Ph	H	PhSO_2_	toluene, 100 °C, 24 h	25:75	56	[68]
32	CH_2_CH_2_CH_2_		Ph	xylene	≈75:25	51/18	[69]
33	Ph	I	COOMe	xylene, reflux, 24 h	58:42	n.d.	[20]
35	2-Et-Ph	I	COOMe	xylene, reflux, 24 h	56:44	n.d.	[20]
36	Me	H	COOMe	toluene, reflux, 12 h	100:0	75	[70]
37	CH_2_CH_2_CH_2_CH_2_		COOMe	xylene, reflux, 10 h xylene, reflux, 16 h xylene, reflux, 6 h	67:33 n.d. n.d.	60 65/26 56/–	[71] [77] [83]
38	CH_2_CH_2_CH_2_CH_2_		COOEt	xylene, reflux, 10 h	75:25	75	[71]
39	CH_2_CH_2_CH_2_CH_2_		COO*n*-Bu	xylene, reflux, 10 h	63:37	72	[71]
40	CH_2_CH_2_CH_2_CH_2_		COOBn	xylene, reflux, 10 h	69:31	59	[71]
41	CH_2_CH_2_CH_2_CH_2_		COO(1-PhEt)	xylene, reflux, 10 h	66:34	60	[71]
42	4-Br-2-Et-Ph	I	COOEt	xylene, reflux, 24 h	–	–	[24]
43	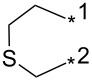		COOMe	*o*-xylene, reflux, 15 h	n.d.	68/12	[72,77]
44	CH_2_SCH_2_		COOMe	*o*-xylene, reflux, 19 h xylene, reflux, 4 h	n.d.	49/22 53/21	[72–73] [36]
45	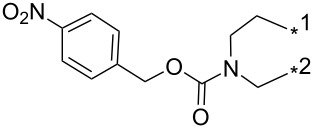		COOMe	*o*-xylene, reflux, 16 h	n.d.	64/24	[72,77]
46	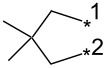		COOMe	*o*-xylene, reflux, 16 h	n.d.	32/32	[72]
47	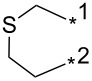		COOMe	*o*-xylene, reflux, 16 h *o*-xylene, reflux, 15 h	n.d.	59/34 68/12	[72] [73]
48	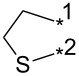		COOMe	*o*-xylene, reflux, 21 h	40:60	80	[72]
49	CH_2_CH_2_CH_2_		COOMe	xylene, reflux, 8 h 1,2-diethoxyethane, 120–125°C, 8 h	n.d. ≈87:13	40/35 47	[13] [74]
50	4-EtO-Ph	H	COOEt	chlorobenzene, reflux, 48 h	76:24	90	[75]
51	4-EtO-Ph	I	COOEt	chlorobenzene, reflux, 48 h	56:44	81	[75]
52	4-EtO-Ph	CN	COOEt	chlorobenzene, reflux, 48 h	58:42	80	[75–76]
53	4-EtO-Ph	CH_2_OH	COOEt	chlorobenzene, reflux, 48 h	63:37	71	[75]
54	4-EtO-Ph	PhS	COOEt	chlorobenzene, reflux, 48 h	52:48	71	[75]
55	4-EtO-Ph	CN	COOBn	chlorobenzene, reflux, 48 h	57:43	76	[75–76]
56	4-EtO-Ph	CN	COO*t*-Bu	chlorobenzene, reflux, 48 h	78:22	79	[75–76]
57	4-EtO-Ph	CN	COOCHPh_2_	chlorobenzene, reflux, 48 h	100:0	85	[75–76]
56a	Ph	CN	COOCHPh_2_	chlorobenzene, reflux, 48 h	100:0	80	[75–76]
57a	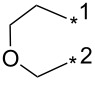		COOMe	*o*-xylene, reflux, 21 h	n.d.	87	[77]
58	2,3-diMe-Ph	H	COOMe	xylene, reflux, 12 h	75:25	n.d.	[28]
59	Ph	H	Me_3_Si	toluene, reflux	n.d.	95/–	[78]
60	Ph	H	Me_2_PhSi	toluene, reflux	n.d.	80/–	[78]
61	Ph	H	*t*-BuPh_2_Si	toluene, reflux	n.d.	15/–	[78]
62	Ph	H	BPin	mesitylene, reflux, 16 h	88:12	47/7	[79,81]
63	Ph	Ph	4-(Me_2_N)-Ph	*o*-DCB, reflux, 48 h	n.d.	65/–	[80]
64	4-NO_2_-Ph	Me	BPin	*o*-DCB, reflux, 24 h	89:11	79	[81]
65	4-NO_2_-Ph	iPr	BPin	*o*-DCB, reflux, 24 h	>98:2	75	[81]
66	CH_2_CH_2_CH_2_CH_2_		BPin	xylene, reflux, 24 h	90:10	78	[81]
67	4-NO_2_-Ph	H	Ph	xylene, 140 °C, 8 h	95:5	60	[82]
68	4-NO_2_-Ph	I	Ph	xylene, 140 °C, 8 h	91:9	84	[82]
69	Ph	I	Ph	xylene, 140 °C, 16 h	>95:5	73	[82]
70	4-MeO-Ph	H	Ph	xylene, 140 °C, 24 h *o*-DCB, 140 °C, 24 h	91:9 91:9	30 76	[82] [92]
71	4-MeO-Ph	I	Ph	xylene, 140 °C, 24 h	91:9	72	[82]
72	4-NO_2_-Ph	H	Me_3_Si	xylene, 140 °C, 8 h	89:11	75^b^	[82]
73	4-NO_2_-Ph	I	Me_3_Si	xylene, 140 °C, 8 h	95:5	99^b^	[82]
74	4-NO_2_-Ph	H	*n*-Bu	xylene, 140 °C, 8 h	91:9	47^b^	[82]
75	4-NO_2_-Ph	I	*n*-Bu	xylene, 140 °C, 8 h	91:9	82^b^	[82]
76	4-NO_2_-Ph	I	Bn	xylene, 140 °C, 8 h	91:9	64^b^	[82]
77	4-NO_2_-Ph	I	cyclo-Pr	xylene, 140 °C, 8 h	94:6	77^b^	[82]
78	4-NO_2_-Ph	I	CH_2_OBn	xylene, 140 °C, 8 h	>95:5	62^b^	[82]
79	4-NO_2_-Ph	I	C(OH)Ph_2_	xylene, 140 °C, 8 h	>95:5	70^b^	[82]
80	4-NO_2_-Ph	I	4-MeO_2_C-Ph	xylene, 140 °C, 8 h	>95:5	65^b^	[82]
81	3-Py	H	Ph	*o*-DCB, μ-w, 200 °C, 2 h	89:11	84	[84]
82	Ph	H	2-Py 2-PyH^+^ TsO^–^	*o*-DCB, μ-w, 200 °C, 2 h ethylene glycol, reflux, 16 h	60:40 91:9	85 14	[84]
83	3-Py	H	2-Py	*o*-DCB, μ-w, 200 °C, 2 h	67:33	80	[84]
84	Ph	H	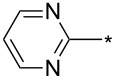	*o*-DCB, μ-w, 200 °C, 2 h	60:40	86	[84]
85	3-Py	H	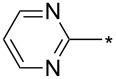	*o*-DCB, μ-w, 200 °C, 2 h	60:40	84	[84]
86	4-NO_2_-Ph	Me	2-Py	*o*-DCB, reflux, 20 h	80:20	87	[84]
87	4-NO_2_-Ph	iPr	2-Py	*o*-DCB, reflux, 20 h	86:24	78	[84]
88	4-NO_2_-Ph	iPr	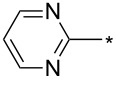	*o*-DCB, reflux, 20 h	n.d.	66/19	[84]
89	3-Py	H	BPin	mesitylene, reflux, 16 h	89:11	84	[84]
90	CH_2_CH_2_CH_2_		4-F-Ph	mesitylene, 155–160 °C, 16 h	n.d.	27/–	[85]
91	Ph	CF_3_	Ph	*o*-DCB, 180 °C, 24 h	94:6	87	[32–33]
92	Ph	CF_3_	cyclo-Pr	*o*-DCB, 180 °C, 24 h	>98:2	88	[32–33]
93	Ph	CF_3_	Me_3_Si	*o*-DCB, 180 °C, 24 h	>98:2	75	[32–33]
94	Ph	CF_3_	2-Py	*o*-DCB, 180 °C, 24 h	95:5	84	[32–33]
95	Ph	CF_3_	BnOCH_2_	*o*-DCB, 180 °C, 24 h	96:4	84	[32–33]
96	Ph	CF_3_	2-F-4-Cl-5-Me-Ph	*o*-DCB, 180 °C, 24 h	n.d.	86/–	[32]
97	Ph	CF_3_	Bu	*o*-DCB, 180 °C, 24 h	>98:2	78	[33]
98	Ph	CF_3_	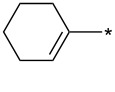	*o*-DCB, 180 °C, 24 h	>98:2	89	[33]
99	Ph	CF_3_	(CH_2_)_3_Cl	*o*-DCB, 180 °C, 24 h	98:2	70	[33]
100	4-MeO-Ph	CF_3_	Ph	*o*-DCB, 180 °C, 24 h	>98:2	85	[33]
101	4-MeO-Ph	CF_3_	Bu	*o*-DCB, 180 °C, 24 h	>98:2	71	[33]
102	4-MeO-Ph	CF_3_	cyclo-Pr	*o*-DCB, 180 °C, 24 h	>98:2	75	[33]
103	4-NO_2_-Ph	CF_3_	Ph	*o*-DCB, 180 °C, 24 h	>98:2	85	[33]
104	4-NO_2_-Ph	CF_3_	(CH_2_)_3_Cl	*o*-DCB, 180 °C, 24 h	>98:2	68	[33]
105	Me	CF_3_	Ph	*o*-DCB, 180 °C, 24 h	98:2	95	[33]
106	Me	CF_3_	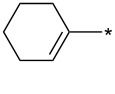	*o*-DCB, 180 °C, 24 h	98:2	82	[33]
107	Me	CF_3_	BnOCH_2_	*o*-DCB, 180 °C, 24 h	>98:2	89	[33]
108	Me	CF_3_	COOEt	*o*-DCB, 180 °C, 24 h	93:7	94	[33]
109	Ph	CF_3_	BPin	*o*-DCB, 140 °C, 72 h	93:7	69	[32–33]
110	Me	CF_3_	BPin	*o*-DCB, 140 °C, 72 h	96:4	44	[33]
111	Bn	CF_3_	Ph (2 equiv) Ph (2 equiv) Ph (2 equiv) Ph (10 equiv)	*o*-DCB, 180 °C, 24 h *o*-DCB, 140 °C, 24 h *o*-DCB, 140 °C, 48 h *o*-DCB, 180 °C, 24 h	64:36 96:4 88:12 88:12	61 34 66 64	[33]
112	Bn	CF_3_	Bu	*o*-DCB, 180 °C, 24 h	72:28	48	[33]
113	Ph	CH_2_OH	Ph	*o*-DCB, 180 °C, 24 h	n.d.	72/–	[33]
114	Ph	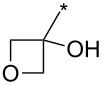	COOEt	*o*-DCB, 180 °C, 30 min, μ-wave	88:12	66	[34]
115	Ph	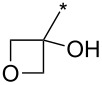	Ph	xylene, 140 °C, 6 h, μ-wave	98:2	51	[34]
116	Ph	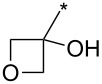	Me_3_Si	xylene, 140 °C, 3.5 h, μ-wave	98:2	17	[34]
117	4-MeO-Ph	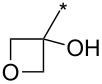	COOEt	*o*-DCB, 180 °C, 1 h, μ-wave	83:17	44	[34]
118	Bn	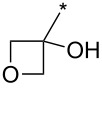	COOEt	*o*-DCB, 180 °C, 30 min, μ-wave	67:33	21	[34]
119	Ph	H	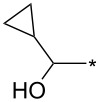	toluene, reflux, 12 h	100:0	33	[86]
120	4-Me-Ph	H	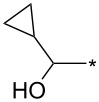	toluene, reflux, 12 h	100:0	35	[86]
121	4-I-Ph	H	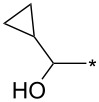	toluene, reflux, 12 h	100:0	40	[86]
122	4-Cl-Ph	H	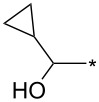	toluene, reflux, 12 h	100:0	43	[86]
123	4-F-Ph	H	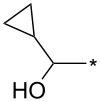	toluene, reflux, 12 h	100:0	38	[86]
124	4-MeO-Ph	H	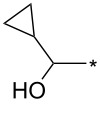	toluene, reflux, 12 h	100:0	33	[86]
125	3,4-OCH_2_O-Ph	H	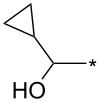	toluene, reflux, 12 h	100:0	30	[86]
126	Ph	H	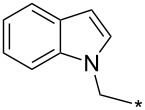	toluene, reflux, 12 h	≈34:67	18/36	[86]
127	4-Me-Ph	H	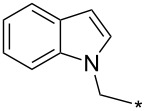	toluene, reflux, 12 h	≈40:60	28/42	[86]
128	4-I-Ph	H	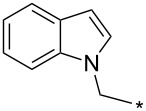	toluene, reflux, 12 h	≈80:20	20/5	[86]
129	4-Cl-Ph	H	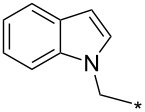	toluene, reflux, 12 h	≈83:17	35/4	[86]
130	4-F-Ph	H	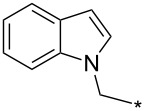	toluene, reflux, 12 h	≈67:33	20/10	[86]
131	4-MeO-Ph	H	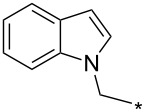	toluene, reflux, 12 h	≈20:80	10/40	[86]
132	3,4-OCH_2_O-Ph	H	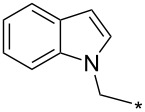	toluene, reflux, 12 h	≈67:33	20/10	[86]
133	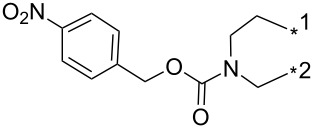		CF_3_	*o*-xylene, −78–270 °C, 12 h	n.d.	61/10	[87]
134	Ph	H	3,5-di-HC≡C-Ph	*N*-methylpyrrolidone, 185 °C, 48 h	n.d.	(32)	[88]
135	3,4,5-tri-MeO-Ph	3-BnO-4-MeO-Ph	Me_3_Si	xylene, 160 °C, 24 h	95:5	74	[89]
136	3-BnO-4-MeO-Ph	3,4,5-tri-MeO-Ph	Me_3_Si	xylene, 160 °C, 24 h	95:5	79	[89]
137	Me	H	3,4,5-tri-MeO-Ph	xylene, 160 °C, 24 h	>98:2	88	[89]
138	Bn	H	3,4,5-tri-MeO-Ph	xylene, 160 °C, 24 h	90:10	65	[89]
139	Me	H	3-TBSO-4-MeO-Ph	xylene, 160 °C, 24 h	90:10	65	[89]
140	Bn	H	3-TBSO-4-MeO-Ph	xylene, 160 °C, 24 h	90:10	58	[89]
141	4-MeO-Ph	4-MeO-Ph	COOEt	*o*-DCB, 140–180 °C, 16 h	50:50	<60	[90]
142	4-MeO-Ph	4-MeO-Ph	3-CN-4-Cl-Ph-CO	*o*-DCB, 140 °C, 16 h	50:50	95	[90]
143	4-MeO-Ph	4-MeO-Ph	CH_2_OH	xylene, 160 °C, 24 h	93:7	97	[90]
144	4-MeO-Ph	3,4,5-tri-MeO-Ph	Me_3_Si	xylene, 160 °C, 24 h	95:5	100	[91]
145	3,4,5-tri-MeO-Ph	3-NH_2_-4-MeO-Ph	Me_3_Si	xylene, 160 °C, 24 h	95:5	91	[91]
146	Ph	CON(Me)OMe	Me_3_Si	xylene, 160 °C, 24 h	95:5	89	[91]
147	Bn	3-CF_3_-Ph	Me_3_Si	xylene, 160 °C, 24 h	95:5	77	[91]
148	3,4,5-tri-MeO-Ph	3-OH-4-MeO-Ph	Me_3_Si	xylene, 160 °C, 24 h	95:5	82	[91]
149	Ph	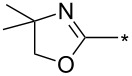	Me_3_Si	xylene, 160 °C, 24 h	95:5	87	[91]
150	4-F-Ph	H	Ph	xylene, 160 °C, 24 h	90:10	100	[91]
151	4-MeO-Ph	H	cyclo-Pr	xylene, 160 °C, 24 h	90:10	91	[91]
152	Ph	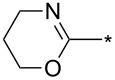	cyclo-Pr	xylene, 160 °C, 24 h	95:5	91	[91]
153	Ph	4-Me-Ph	CH_2_OH	xylene, 160 °C, 24 h	95:5	63	[91]
154	Ph	CON(Me)OMe	cyclo-Pr	xylene, 160 °C, 24 h	95:5	84	[91]
155	Ph	2-Py	Ph	xylene, 160 °C, 24 h	95:5	98	[91]
156	Ph	H	COOEt	*o*-DCB, 140 °C, 16 h	67:33	59	[92]
156	4-MeO-Ph	H	COOEt	*o*-DCB, 140 °C, 24 h	67:33	57	[92]

^a^Isolated yield of single or both regioisomers. ^b^In a sealed tube. n.d. – not determined.

**Scheme 7:**
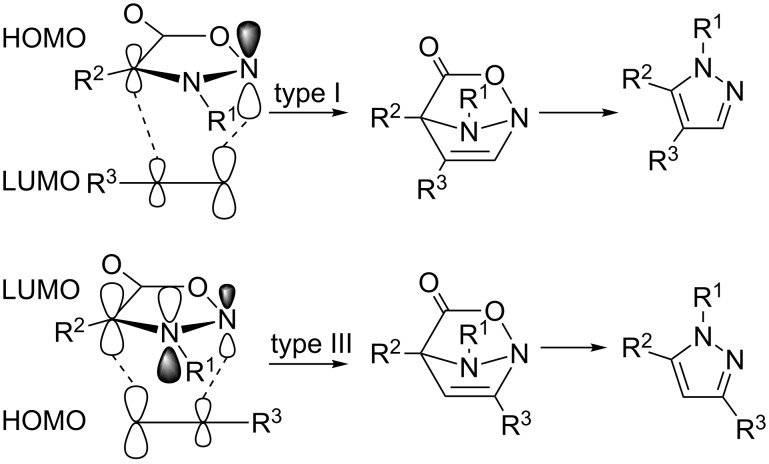
HOMO–LUMO diagram for thermal [3 + 2]-cycloaddition of sydnones with alkynes.

The first people who qualitatively discussed the regioselectivity on the basis of semi-empirical quantum calculations was Houk et al*.* [94] who (except of above-mentioned low-lying LUMO of sydnone [58]) calculated sydnone LUMO terminal orbital coefficients and found them to be almost identical thus indicating low selectivity in LUMO-controlled cycloadditions (type III). However, Gotthardt and Reiter [8] who were also dealing with regioselectivity of sydnone cycloadditions with methyl propiolate pointed out that the reason for the lower regioselectivity can also be attributed to the low-lying HOMO of this dipolarophile. While for the LUMO-controlled reaction (type III) only the 3-substituted pyrazole is expected to be the main product, for the HOMO-controlled reaction (type I) 4-substituted pyrazole should be formed preferentially (Scheme 7 adapted from reference [8]).

The combination of both reaction pathways (type II) therefore gives a mixture of 3- and 4-substituted pyrazoles. This situation is typical especially for cycloadditions with alkyl propiolates (cf. Table 4, entries 6, 7, 23–29, 33, 34, 50–55, 58, 118, 141) and acylalkynes (Table 4, entry 142). Other terminal alkynes, for which the calculated lower HOMO–LUMO energy gaps correspond to the type III mechanism (especially phenylacetylenes, alkylacetylenes, trimethylsilylacetylene and BPin-acetylene), innately prefer formation of 1,3- (or 1,3,5-) di- (or tri-)substituted pyrazoles in ratios about or even better than 90:10. Recent quantum calculations undertaken for 3-phenylsydnone and phenylacetylene by Harrity et al. [92] clearly support a preferential formation of 1,3-diphenyl-1*H*-pyrazole. The calculated difference in energies of transition states leading to 1,3- and 1,4-diphenyl-1*H*-pyrazole (4.1 kcal·mol^−1^ = 17.2 kJ·mol^−1^) predicts at 140 °C the ratio ≈99:1 which corresponds well with found experimental value >95:5 (see entry 3 in Table 4 [83]).

There are only some known exceptions in which the 1,4-substituted pyrazole prevails (Table 4, entries 8, 23, 31, 48, 126, 127 and 131). The most significant is the reaction of the parent phenylsydnone with phenylsulfonylacetylene which gives the ratio 25:75 [68] consistent with the strong electron-withdrawing effect of the phenylsulfonyl group lowering the HOMO of this dipolarophile. In addition, quantum calculations of orbital coefficients show that the HOMO is mainly located on the phenyl moiety and not in the acetylene moiety thus excluding the type III mechanism leading to a 1,3-disubstituted pyrazole.

Even though the HOMO–LUMO energy gaps and terminal orbital coefficients can be tuned by substitution of the sydnone (and alkyne) the ratio of both isomers is often only slightly influenced. For example 3-(substituted phenyl)sydnones react with methyl propiolate to give a mixture of both regioisomers in a 75:25 ratio (for 2,3-diMe [28], 4-OEt [75], H [1–2]), whilst only for 4-NO_2_ derivative [8] is an enhanced ratio of 86:14 observed. The same pattern can be seen for reaction of 3,4-diphenylsydnones. While unsubstituted (R^1^ = R^2^ = Ph) reacts with methyl propiolate to give equimolar amounts of the corresponding pyrazole 3-/4-carboxylate, an introduction of one or two nitro group(s) into position(s) 4- or 2,4- of the 3-phenyl ring (R^1^ = 4-NO_2_-Ph or 2,4-diNO_2_-Ph) leads to ratios 56:44 and 61:39, respectively [8]. The presence of the nitro group(s) lower(s) the LUMO energy of the sydnone and a type III mechanism is slightly favored. The same trend [8] can be seen from the substitution effect in position 4 of the starting 3-phenylsydnone when reacted with methyl propiolate (Table 4, entries 6, 7, 23–26) but almost no influence is observed for reactions with phenylacetylene (Table 4, entries 3, 4, 69, 91). Generally, it can be concluded that any substituent in position 4 reduces the regioselectivity.

The steric hindrance can also affect the ratio of the regioisomers formed. The classical example was described by Yeh et al. [75–76] who performed reactions of 3-(4-ethoxyphenyl)sydnone-4-carbonitrile with various alkyl propiolates (Et, Bn, *t*-Bu, Ph_2_CH – see entries 52, 55–57 in Table 4) and observed that the 3-/4-ratio increased from 58:42 to 100:0. However, this trend is not general because Lee et al. [71] observed the best regioselectivity for the reaction of 4,5,6,7-tetrahydro[1,2,3]oxadiazolo[3,4-*a*]pyridin-8-ium-3-olate with methyl propiolate and the lowest selectivity with *n*-butyl and 1-phenylethyl propiolates (see entries 38–41 in Table 4).

The last factor that influences the ratio of isomers involves the thermodynamic conditions – namely the temperature and pressure. A nice temperature/pressure-selectivity study of the cycloaddition of 3-phenylsydnone with methyl propiolate was undertaken by McGowin et al. [93] in supercritical CO_2_. At 7.6 MPa they found a linear dependence between the natural logarithm of selectivity (defined as the 3-/4-isomer ratio) and the reaction temperature. In accordance with the common reactivity–selectivity principle, the higher temperature lowers selectivity from 5.52 (i.e*.,* 85:15) at 80 °C to 3.14 (i.e., 76:24) at 160 °C but increases sydnone conversion and pyrazole yield. On the other hand, a variation of the pressure from 7.6 to 30.4 MPa at constant temperature (80 °C) caused a decrease in the total yield by approximately 50%, with slightly increased selectivity (from 4.96 to 6.56). Lowering of the yield with increasing pressure confirms the reversibility of the first step (see Scheme 5) because of retardation of CO_2_ cleavage from the bicyclic intermediate. Such reversibility was also suggested by Harrity et al. [92] on the basis of quantum calculations. While the formation of the bicyclic intermediate was calculated to be only slightly exergonic (−3.3 kcal·mol^−1^) the overall reaction is highly exothermic (−108.2 kcal·mol^−1^).

These results show that minor differences in selectivity published by various authors (e.g., entry 3 in Table 4) can be ascribed to changes in temperature (different boiling point of benzene, toluene, xylenes, DCB, …) used in synthesis. In several cases (e.g., entries 83-86 [84] and 141 and 142 [90] in Table 4) too high temperature (200 °C) can contribute to a substantial drop of selectivity. It is also known that some sydnones start to decompose at temperatures exceeding 180 °C [74] which can cause lowering of the pyrazole yield.

**Scheme 8:**
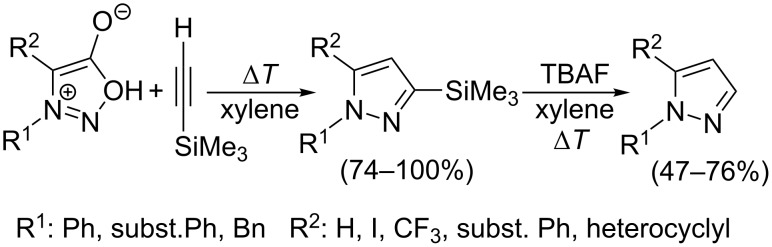
Synthetic strategy leading to 1,2-disubstituted pyrazoles.

From a synthetic point of view, the cycloaddition with terminal alkynes represents a very good strategy for the preparation of 1-,1,3-, 1,5- and 1,3,5-substituted pyrazoles. Although 1- and 1,5-(di)substituted pyrazoles are directly available from 3- or 3,4-(di)substituted sydnones and acetylene (e.g., entry 1 in Table 1 [1–2]), handling with gaseous acetylene or its solution in pressurized reaction vessels is inconvenient and may be even dangerous. Two strategies can overcome such problems: liquid DMAD, diethyl acetylenedicarboxylate or alkyl propiolate can be used instead of acetylene and the resulting pyrazole-3,4-dicarboxylates or pyrazole-3-/4-carboxylates can then undergo hydrolysis and decarboxylation [16,95–96]. A novel strategy (Scheme 8) was recently developed by Harrity et al. (see entries 73, 74, 94, 117, 136, 137, 145–150 [32–34,82,89,91] in Table 4) who used trimethylsilyl acetylene as a dipolarophile. After regioselective cycloaddition giving 3-trimethylsilylpyrazole (cf. also entries 59–61 [78]) in high yields (74–100%) the trimethylsilyl group was removed by TBAF-mediated protodesilylation in moderate to good yields (47–76%).

### Thermal reaction of sydnones with internal non-symmetrical alkynes and cycloakynes

**Scheme 9:**
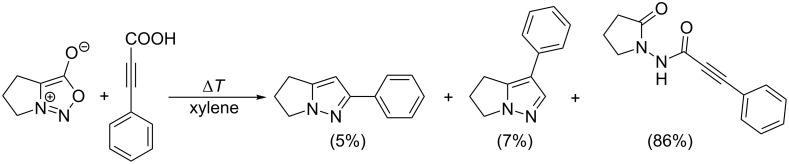
Unsuccessful reaction with phenylpropiolic acid.

The reaction with internal non-symmetrical alkynes giving 1,3,4-trisubstituted or even 1,3,4,5-tetrasubstituted pyrazoles seems to be the most complicated case of cycloaddition due to the influence of both substituents (R^3^, R^4^) bound on a triple bond on the formation of pyrazole regioisomers. Moreover, not all substituents are compatible with the reaction conditions. For example phenylpropiolic acid (R^3^: COOH) gives only minor a yield of the cycloaddition/decarboxylation product with 5,6-dihydro-3-hydroxy-4*H*-pyrrolo[1,2-*c*][1,2,3]oxadiazol-7-ium [97–98] (Scheme 9).

The Table 5 again summarizes all the examples found, including reaction conditions from which we have come to several conclusions.

**Table 5:** Thermal cycloaddition of sydnones with internal non-symmetrical alkynes.

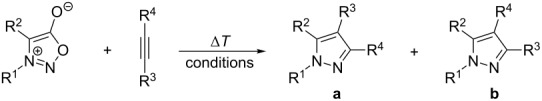								

entry	R^1^	R^2^	R^3^	R^4^	conditions	ratio a:b	yield [%]^a^	ref.

1	Ph	H	Me	Ph	xylene, 135–140 °C, 20 h	n.d.	83/–	[1–2]
2	Ph	H	COOEt	Ph	toluene, 95 °C, 84 h	n.d.	82–83/–	[1–2]
3	4-Cl-Ph	H	COOEt	Ph	xylene, reflux, 3 h	n.d.	92/–	[2]
4	4-MeO-Ph	H	COOEt	Ph	xylene, reflux, 3 h	n.d.	83/–	[2]
5	4-Me-Ph	H	COOEt	Ph	xylene, reflux, 3 h	n.d.	98/–	[2]
6	Bn	H	COOEt	Ph	xylene, reflux, 16 h	n.d.	46/–	[2]
7	Ph	Ph	COOEt	Ph	*p*-cymene, 160 °C, 16 h	n.d.	87/–	[2]
8	Ph	Me	COOEt	Ph	xylene, 110 °C, 8 h	100:0	82	[1–2]
9	Ph	H	COMe	Ph	chlorobenzene, 130 °C, 12 h	100:0	100	[1–2]
10	Ph	H	COPh	Ph	xylene, 135–140 °C, 16 h	100:0	82	[1–2]
11	Me	H	COPh	Ph	*o*-DCB, reflux, 144 h	69:31	99	[16]
12	Ph	H	CN	Cl	chlorobenzene, 110 °C, 10 h	n.d.	15/20	[66]
13	Ph	H	SO_2_Ph	Me	toluene, 100 °C, 24 h	100:0	58	[68]
14	Ph	H	SO_2_Ph	Ph	toluene, 100 °C, 24 h	100:0	73	[68]
15	Ph	H	COOMe	CH(OMe)_2_	toluene, reflux, 60 h	21:79	84	[99]
16	Bn	H	COOMe	CH(OMe)_2_	toluene, reflux, 72 h	19:81	80	[99]
17	Bn	H	COOMe	CHO	toluene, reflux, 18 h	72:28	90	[99]
18	Ph	H	COOMe	CHO	toluene, reflux, 18 h	66:34	93	[99]
19	Bn	H	COOMe	CH_2_OH	toluene, reflux, 72 h	50:50	75	[99]
20	Ph	H	COOMe	CH_2_OH	toluene, reflux, 48 h	60:40	79	[99]
21	Ph	H	CF_3_	4-MeO-Ph	xylene, 120 °C, 48–72 h	93:7	56	[100]
22	Ph	H	CF_3_	4-NO_2_-Ph	xylene, 120 °C, 48–72 h	93:7	93	[100]
23	Ph	H	CF_3_	4-MeS-Ph	xylene, 120 °C, 48–72 h	93:7	90	[100]
24	Ph	H	CF_3_	2-Cl-Ph	xylene, 120 °C, 48–72 h	94:6	92	[100]
25	Ph	H	CF_3_	4-MeSO_2_-Ph	xylene, 120 °C, 48–72 h	92:8	86	[100]
26	Ph	H	CF_3_	4-Cl-Ph	xylene, 120 °C, 48–72 h	93:7	75	[100]
27	4-Cl-Ph	H	CF_3_	4-Cl-Ph	xylene, 120 °C, 48–72 h	93:7	90	[100]
28	4-MeO-Ph	H	CF_3_	4-Cl-Ph	xylene, 120 °C, 48–72 h	93:7	84	[100]
29	Bn	H	CF_3_	4-Cl-Ph	xylene, 120 °C, 48–72 h	91:9	65	[100]
30	*t*-Bu	H	CF_3_	4-Cl-Ph	xylene, 120 °C, 48–72 h	93:7	58	[100]
31	Me	H	CF_3_	4-Cl-Ph	xylene, 120 °C, 48–72 h	92:8	92	[100]
32	Ph	Me	CF_3_	4-Cl-Ph	xylene, 120 °C, 48–72 h	84:16	75	[100]
33	Ph	4-Cl-Ph	CF_3_	4-Cl-Ph	xylene, 120 °C, 48–72 h	60:40	57	[100]
34	Ph	Br	CF_3_	4-Cl-Ph	xylene, 120 °C, 48–72 h	71:29	73	[100]
35	Ph	MeS	CF_3_	4-Cl-Ph	xylene, 120 °C, 48–72 h	43:57	62	[100]
36	*t*-Bu	H	COOEt	Et	xylene, reflux, 72 h	n.d.	38/8	[101]
37	Ph	H	PhCO	5-NO_2_-furan-2-yl	xylene, reflux, 3–4 h	100:0	74	[102]
38	Ph	H	4-Me-PhCO	5-NO_2_-furan-2-yl	xylene, reflux, 3–4 h	100:0	80	[102]
39	Ph	H	4-Cl-PhCO	5-NO_2_-furan-2-yl	xylene, reflux, 3–4 h	100:0	72	[102]
40	4-MeO-Ph	H	PhCO	5-NO_2_-furan-2-yl	xylene, reflux, 3–4 h	100:0	73	[102]
41	4-MeO-Ph	H	4-Me-PhCO	5-NO_2_-furan-2-yl	xylene, reflux, 3–4 h	100:0	74	[102]
42	4-MeO-Ph	H	4-Cl-PhCO	5-NO_2_-furan-2-yl	xylene, reflux, 3–4 h	100:0	73	[102]
43	4-Me-Ph	H	PhCO	5-NO_2_-furan-2-yl	xylene, reflux, 3–4 h	100:0	79	[102]
44	4-Me-Ph	H	4-Me-PhCO	5-NO_2_-furan-2-yl	xylene, reflux, 3-4 h	100:0	83	[102]
45	4-Me-Ph	H	4-Cl-PhCO	5-NO_2_-furan-2-yl	xylene, reflux, 3–4 h	100:0	75	[102]
46	Me	H	COOEt	CF_3_	xylene, 100 °C, 4 h	n.d.	18/25	[103]
47	Ph	H	SnBu_3_	SiMe_3_	toluene, reflux	100:0	80	[78]
48	Ph	H	SiMe_2_Ph	SiMe_3_	toluene, reflux	n.d.	63/34	[78]
49	Ph	H	COMe	SiMe_3_	toluene, reflux	n.d.	81/16	[78]
50	Ph	H	BPin	Ph	xylene, reflux, 4 h	98:2	58	[79,81]
51	Ph	H	BPin	Bu	xylene, reflux, 4 h	71:29	64	[79,81]
52	Ph	H	BPin	Me_3_Si	xylene, reflux, 4 h	67:33	76	[79,81]
53	4-MeO-Ph	H	BPin	Ph	xylene, reflux, 4 h	98:2	58	[79]
54	4-NO_2_-Ph	H	BPin	Ph	xylene, reflux, 4 h	98:2	70	[79]
55	4-MeO-Ph	H	BPin	Bu	xylene, reflux, 4 h	83:17	55	[79]
56	4-NO_2_-Ph	H	BPin	Bu	xylene, reflux, 4 h	83:17	62	[79]
57	4-MeO-Ph	H	BPin	Me_3_Si	xylene, reflux, 4 h	67:33	61	[79]
58	4-NO_2_-Ph	H	BPin	Me_3_Si	xylene, reflux, 4 h	60:40	83	[79]
59	3-Py	H	BPin	Ph	xylene, reflux, 16 h	>98:2	60	[84]
60	3-Py	H	BPin	Me_3_Si	xylene, reflux, 16 h	57:43	70	[81,84]
61	3-Py	H	BPin	*n*-Bu	mesitylene, reflux, 16 h	71:29	56	[84]
62	4-Me-Ph	CHO	PhCO	5-NO_2_-furan-2-yl	xylene, reflux, 3–4 h	n.d.	79/–	[104]
63	4-Me-Ph	CHO	4-Me-PhCO	5-NO_2_-furan-2-yl	xylene, reflux, 3–4 h	n.d.	74/–	[104]
64	4-Me-Ph	Br	4-MeO-PhCO	5-NO_2_-furan-2-yl	xylene, reflux, 3–4 h	n.d.	62/–	[104]
65	4-MeO-Ph	Br	4-MeO-PhCO	5-NO_2_-furan-2-yl	xylene, reflux, 3–4 h	n.d.	69/–	[104]
66	4-Me-Ph	Br	PhCO	5-NO_2_-furan-2-yl	xylene, reflux, 3–4 h	n.d.	73/–	[104]
67	4-Me-Ph	Br	4-Me-PhCO	5-NO_2_-furan-2-yl	xylene, reflux, 3–4 h	n.d.	66/–	[104]
68	4-MeO-Ph	Br	4-Me-PhCO	5-NO_2_-furan-2-yl	xylene, reflux, 3–4 h	n.d.	63/–	[104]
69	Ph	Br	4-Me-PhCO	5-NO_2_-furan-2-yl	xylene, reflux, 3–4 h	n.d.	72/–	[104]
70	4-MeO-Ph	MeCO	PhCO	5-NO_2_-furan-2-yl	xylene, reflux, 3–4 h	n.d.	74/–	[104]
71	4-MeO-Ph	MeCO	4-MeO-PhCO	5-NO_2_-furan-2-yl	xylene, reflux, 3–4 h	n.d.	73/–	[104]
72	Ph	H	4-Me-PhCO	5-NO_2_-thiophen-2-yl	xylene, reflux, 3–4 h	n.d.	71/–	[105]
73	Ph	H	4-MeO-PhCO	5-NO_2_-thiophen-2-yl	xylene, reflux, 3–4 h	n.d.	73/–	[105]
74	4-Me-Ph	H	PhCO	5-NO_2_-thiophen-2-yl	xylene, reflux, 3–4 h	n.d.	75/–	[105]
75	4-Me-Ph	H	4-Me-PhCO	5-NO_2_-thiophen-2-yl	xylene, reflux, 3–4 h	n.d.	73/–	[105]
76	Ph	H	4-Cl-PhCO	5-NO_2_-thiophen-2-yl	xylene, reflux, 3–4 h	n.d.	72/–	[105]
77	4-Me-Ph	H	4-Cl-PhCO	5-NO_2_-thiophen-2-yl	xylene, reflux, 3–4 h	n.d.	77/–	[105]
78	4-MeO-Ph	H	4-Cl-PhCO	5-NO_2_-thiophen-2-yl	xylene, reflux, 3–4 h	n.d.	78/–	[105]
79	Ph	H	PhCO	5-NO_2_-thiophen-2-yl	xylene, reflux, 3–4 h	n.d.	80/–	[105]
80	4-MeO-Ph	H	PhCO	5-NO_2_-thiophen-2-yl	xylene, reflux, 3–4 h	n.d.	75/–	[105]
81	4-MeO-Ph	H	4-Me-PhCO	5-NO_2_-thiophen-2-yl	xylene, reflux, 3–4 h	n.d.	76/–	[105]
82	CH_2_CH_2_CH_2_		*p*-Tos	Bu	anisol, reflux, 0.5 h	n.d.	90/–	[106]
83	CH_2_CH_2_CH_2_		*p*-Tos	Ph	anisol, reflux, 0.5 h	n.d.	89/–	[106]
84	Ph	4-Me-Ph	BPin	Me_3_Si	*o*-DCB, reflux, 48 h	100:0	48	[80]
85	Ph	4-NO_2_-Ph	BPin	Me_3_Si	*o*-DCB, reflux, 48 h	100:0	70	[80]
86	Me	H	BPin	Ph	mesitylene, reflux, 48 h	>98:2	53	[81]
87	Bn	H	BPin	Ph	xylene, reflux	>98:2	62	[81]
88	Ph	Ph	BPin	Ph	*o*-DCB, reflux, 48 h	>98:2	59	[81]
89	Ph	Ph	BPin	Me_3_Si	*o*-DCB, reflux, 48 h	>98:2	73	[81]
90	Me	Ph	BPin	Me_3_Si	*o*-DCB, reflux, 48 h	>98:2	68	[81]
91	Ph	Me	BPin	Ph	*o*-DCB, reflux, 48 h	>98:2	53	[81]
92	Ph	iPr	BPin	Ph	*o*-DCB, reflux, 48 h	>98:2	38	[81]
93	Ph	Me	BPin	Me_3_Si	*o*-DCB, reflux, 48 h	>98:2	56	[81]
94	Ph	iPr	BPin	Me_3_Si	*o*-DCB, reflux, 48 h	>98:2	43	[81]
95	4-NO_2_-Ph	Me	BPin	Ph	*o*-DCB, reflux, 18 h	>98:2	67	[81]
96	4-NO_2_-Ph	iPr	BPin	Ph	*o*-DCB, reflux, 24 h	>98:2	45	[81]
97	4-NO_2_-Ph	Me	BPin	Me_3_Si	*o*-DCB, reflux, 18 h	>98:2	80	[81]
98	4-NO_2_-Ph	iPr	BPin	Me_3_Si	*o*-DCB, reflux, 24 h	>98:2	69	[81]
99	CH_2_CH_2_CH_2_		BPin	Me_3_Si	xylene, reflux, 24 h *o*-DCB, 180 °C, 24 h	>98:2 100:0	21 66	[81] [107]
100	CH_2_CH_2_CH_2_CH_2_		BPin	Me_3_Si	xylene, reflux, 24 h	>98:2	79	[81]
101	CH_2_CH_2_CH_2_CH_2_		BPin	Ph	xylene, reflux, 24 h	>98:2	70	[81]
102	CH_2_CH_2_CH_2_		BPin	Ph	*o*-DCB, 180 °C, 72 h	50:50	51	[107]
103	CH_2_CH_2_CH_2_CH_2_		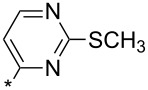	4-F-Ph	mesitylene, 165 °C, 18 h	n.d.	46/–	[108]
104	CH_2_CH_2_CH_2_CH_2_		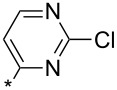	4-F-Ph	mesitylene, 165 °C, 18 h	n.d.	66/–	[108]
105	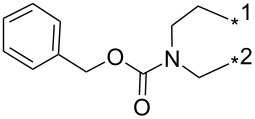		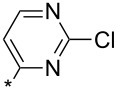	4-F-Ph	mesitylene, 140 °C, 4 h	n.d.	30/–	[108]
106	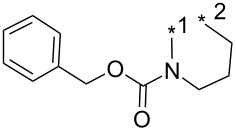		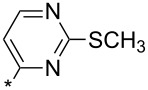	4-F-Ph	mesitylene, 140 °C, 4 h	n.d.	45/–	[108]
107	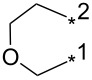		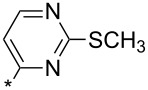	4-F-Ph	mesitylene, 160 °C, 24 h	n.d.	12/–	[108]
108	CH_2_SCH_2_		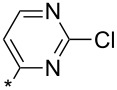	4-F-Ph	mesitylene, 160 °C, 48 h	n.d.	13/–	[108]
109	4-Cl-Ph	H	Me	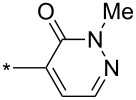	mesitylene, 140 °C, 18 h	n.d.	13/–	[109]
110	Ph	H	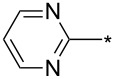	Me_3_Si	*o*-DCB, μ-wave, 200 °C, 2 h	n.d.	55/9	[84]
111	Ph	I	COOEt	Br	toluene, reflux, 18 h	76:24	52/16	[110]
112	Me	I	COOEt	Br	xylene, reflux, overnight	58:42	50/37	[110]
113	Bn	I	COOEt	Br	xylene, reflux, overnight	59:41	39/27	[110]
114	4-F-Ph	I	COOEt	Br	xylene, reflux, overnight	75:25	48/16	[110]
115	4-MeO-Ph	I	COOEt	Br	xylene, reflux, overnight	75:25	63/21	[110]
116	4-MeO-Ph	I	COO*t*-Bu	Br	xylene, reflux, overnight	77:23	43/13	[110]
117	4-MeO-Ph	I	COOEt	I	xylene, reflux, overnight	60:40	58/28	[110]
118	4-MeO-Ph	Br	COOEt	I	xylene, reflux, overnight	–	0/0	[110]
119	CH_2_CH_2_CH_2_CH_2_		4-Py	4-F-Ph	mesitylene, 165 °C, 16 h	n.d.	27/–	[85]
120	Ph	H	COOEt	Br	toluene, reflux, 18 h	46:54	41/48	[111]
121	4-Me-Ph	H	COOEt	Br	toluene, reflux, 18 h	44:56	41/49	[111]
122	4-MeO-Ph	H	COOEt	Br	toluene, reflux, 18 h	41:59	33/49	[111]
123	4-F-Ph	H	COOEt	Br	toluene, reflux, 18 h	47:53	38/43	[111]
124	Ph	CF_3_	COOMe	Me	*o*-DCB, 180 °C, 24 h	85:15	90	[32]
125	Ph	CF_3_	Ph	*n*-Bu	*o*-DCB, 180 °C, 24 h	52:48	62	[32]
126	Ph	CF_3_	BPin	Me_3_Si	*o*-DCB, 140 °C, 48 h	90:10	68	[32]
127	Ph	CF_3_	BPin	*n*-Bu	*o*-DCB, 140 °C, 72 h	>98:2	55	[32]
128	Me	3,4,5- triMeO-Ph	BPin	Me_3_Si	xylene, 180 °C, 24 h	83:17	92	[89]
129	Bn	3,4,5- triMeO-Ph	BPin	Me_3_Si	xylene, 180 °C, 24 h	90:10	66	[89]
130	Me	3-BnO- 4-MeO-Ph	BPin	Me_3_Si	xylene, 180 °C, 24 h	80:20	73	[89]
131	Bn	3-BnO- 4-MeO-Ph	BPin	Me_3_Si	xylene, 180 °C, 24 h	90:10	64	[89]
132	Ph	4-Me-Ph	BPin	Me_3_Si	xylene, 180 °C, 48 h	>98:2	74	[91]
133	Ph	2-Py	BPin	Me_3_Si	xylene, 180 °C, 48 h	>98:2	52	[91]
134	Ph	2-thienyl	BPin	Me_3_Si	xylene, 180 °C, 48 h	88:12	64/6	[91]
135	Me	4-Me-Ph	BPin	Me_3_Si	xylene, 180 °C, 48 h	>98:2	55	[91]
136	4-EtO-Ph	4-MeO-Ph	BPin	Me_3_Si	xylene, 180 °C, 48 h	88:12	74	[91]

^a^Isolated overall yield or isolated yields of both regioisomers a/b. n.d. – not determined.

According to frontier molecular orbital theory both combinations, i.e., HOMO(dipole)–LUMO(dipolarophile) (type I) and HOMO(dipolarophile)–LUMO(dipole) (type III) should lead to the production of individual regioisomers (cf. Scheme 7). All substituents R^1^–R^4^ have an influence on the HOMO–LUMO energy gaps and consequently on the ratio of both isomers especially in those cases when such energy gaps are similar. Again, the substituents on the alkyne (R^3^, R^4^) have great influence on the outcome of the reactions. Strong electron-withdrawing substituents R^3^ (COOR, COR, SO_2_Ar, CF_3_) in combination with any aryl (R^4^: Ph, substituted Ph, heteroaryls) strongly prefer position 4 in the final pyrazole ring (see entries 2–6, 9, 10, 14, 21–28, 37–45, 64–83 in Table 5) when reacting with 4-unsubstituted 3-phenylsydnones or 3-alkylsydnones (see entries 6, 29–31 in Table 5). Both these substituents jointly lower the LUMO while their influence on energy of the HOMO is contradictory. Consequently, the type I mechanism is clearly preferred. If R^4^ has also similar electron-withdrawing ability (e.g., CHO, CF_3_, see entries 17, 18, 46 in Table 5 or R^4^ = halogen, see entries 12, 120–123 in Table 5 and even R^4^ = CH_2_OH, see entries 19 and 20 in Table 5) then almost complete loss of selectivity occurs and the ratio of both regioisomers is close to 50:50. Markedly reversed regioselectivity is observed only for R^4^ = CH(OMe)_2_ which is probably connected with the higher steric demands of this group.

A substitution in position 3 of the sydnone has a much smaller influence on the regioselectivity which is in accordance with longer distance between the substituent and both dipole termini. Substitution of the 3-phenyl ring (e.g., entries 26–28, 37–45, 50, 53, 54 in Table 5) or even change of the whole 3-substituent (alkyls vs phenyl, see entries 29–31, 86, 87 in Table 5) cause no or only a minor change in the ratio of both regioisomers. In some cases the same conclusion can be drawn for changes of the substituent in 4-position of the sydnone (cf. entries 2, 7, 8, 89, 93, 94 or 88, 91, 92 in Table 5). On the other hand, the presence of a substituent can sometimes increase as well as decrease the ratio of both regioisomers (cf. entries 26 and 32–35) for no easily discernible reason.

**Scheme 10:**
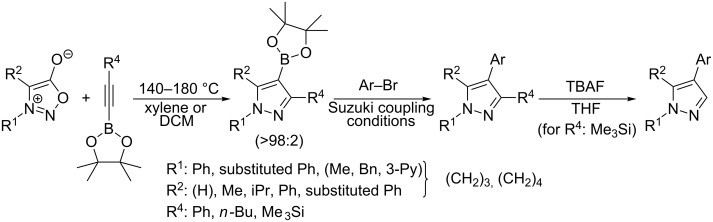
Synthetic strategy leading to 1,4,5-trisubstituted pyrazoles.

A synthetically useful cycloaddition of 4-substituted 3-phenylsydnones with 4,4,5,5-tetramethyl-2-(2-substituted ethynyl)-1,3,2-dioxaborolanes (R^4^–C≡C–BPin; Scheme 10) was recently developed by Harrity et al. [79–81,84,91,107]. In most cases this reaction proceeds with excellent regioselectivity (>98:2) to give the corresponding 1-(substituted phenyl)-3,5-disubstituted -4-BPin-pyrazole, whose BPin group can be easily substituted by an aryl group using a Suzuki–Miyaura cross-coupling reaction. In those cases when a trimethylsilyl group (R^4^) is also present, it can be removed by TBAF-mediated protodesilylation to give a 1,4,5-trisubstituted pyrazole.

It is worth noting that the parent 4,4,5,5-tetramethyl-2-ethynyl-1,3,2-dioxaborolane (R^4^: H) reacts with alkyl/arylsydnones with completely opposite ratio (>7:1) of both regioisomers – i.e., for the BPin group the reaction preferentially occurs in position 3 of the final pyrazole (see entries 62, 64–66, 89, 109 and 110 in Table 4). In this case, using quantum chemical calculations (DFT/B3LYP-6-31G*) [81] steric effects were identified as the main factor influencing the ratio of both regioisomers. These calculations clearly proved the almost apolar character of both possible transition states giving 3- and 4-BPin substituted pyrazoles through bicyclic intermediates (cf. Scheme 5) with a negligible charge transfer flowing from sydnone to the alkyne. This result suggests that there should be a very low influence of the substituents polar effects on the energy of the transition state. Moreover, energy gaps between the dipole HOMO and the dipolarophile LUMO or the dipole LUMO and the dipolarophile HOMO were found to be similar in most cases.

**Scheme 11:**
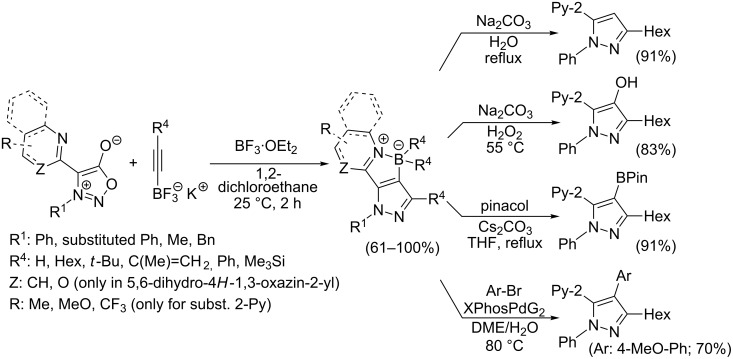
Reaction of sydnones carrying in position 4- six-membered 2-*N*-heterocyclic ring.

Different reaction course was also observed [112] for 3-alkyl and 3-arylsydnones carrying in position 4 a six-membered heterocyclic ring containing a nitrogen atom adjacent to a linkage with parent sydnone ring (pyridin-2-yl, quinolin-2-yl, 5,6-dihydro-4*H*-1,3-oxazin-2-yl). Such sydnones reacted with potassium 2-substituted acetylene trifluoroborates under boron trifluoride diethyl etherate catalysis to give corresponding pyrazolo[3',4':4,5][1,2]azaborolo[2,3-*a*]pyridin-5-ium-4-uides (or quinolin-5-ium-4-uide) in good to excellent yields (Scheme 11). These zwitterionic compounds can be further hydrolyzed to 1,3,5-trisubstituted pyrazoles, oxidized to 4-hydroxy-1,3,5-trisubstituted pyrazoles, transformed to 4-BPin derivative of 1,3,5-trisubstituted pyrazole or arylated under palladium catalysis to give 4-aryl-1,3,5-trisubstitutedpyrazole (Scheme 11). Overall therefore, a nitrogen atom in the sydnone 4-heterocyclyl substituent (especially 2-pyridyl) acts as powerful activating group enabling cycloaddition reaction under ambient conditions and also influencing the regioselectivity. Boron carrying two alkynyl groups always appear formally in position 4 of pyrazole ring including reaction with potassium acetylene trifluoroborate.

**Scheme 12:**
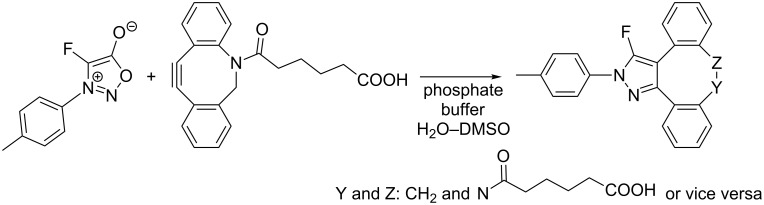
Strain-promoted sydnone alkyne cycloaddition (SPSAC).

The last type of non-symmetrical internal alkynes to be considered are cycloalkynes. Their strain-promoted reactions again proceed quickly under mild reaction conditions (cf. section concerning symmetrical internal alkynes) but their regioselectivity is generally low, which is in accordance with the reactivity–selectivity principle. The first example was described [44] by Taran’s group in 2016 when they observed an ultrafast reaction of 6-[11,12-didehydrodibenzo[*b*,f]azocine-5(6*H*)-yl]-6-oxohexanoic acid with 4-fluoro-3-(4-methylphenyl)sydnone (Scheme 12). Unfortunately the regioselectivity of the reaction was not specified.

**Scheme 13:**
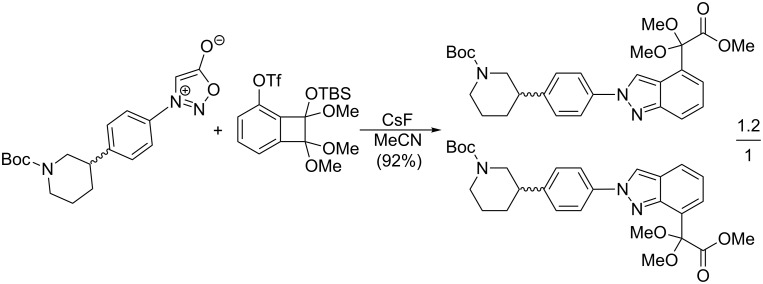
Synthesis of a key intermediate of niraparib.

An aryne generation (Scheme 13) was also used for the synthesis of a key intermediate of the potent antitumor PARP inhibitor – niraparib – containing an indazole core [113]. A substituted 2,3-aryne was generated in situ from (siloxy)benzocyclobutenes and CsF but the regioselectivity was poor: a ratio of both possible regioisomers of 45:55 was obtained.

**Scheme 14:**
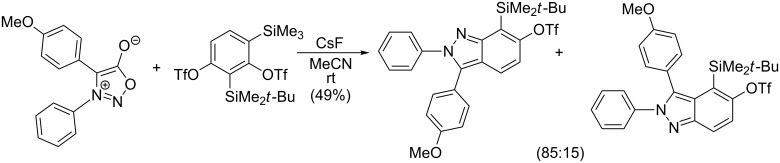
Reaction of sydnones with 1,3-/1,4-benzdiyne equivalents.

A much better regioselectivity was achieved [114] in a reaction of 1,3-/1,4-benzdiyne equivalents (2,4-bissilyl-1,3-bistriflates) with two different dipoles from which one was 3-phenyl-4-(4-methoxyphenyl)sydnone (Scheme 14). The reason for the much better regioselectivity probably lies in the steric hindrance between the bulky *t*-BuMe_2_Si and 4-MeO-Ph groups.

**Scheme 15:**
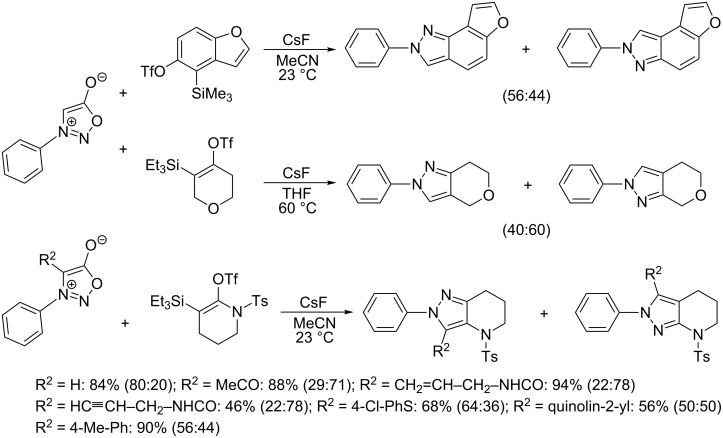
Reaction of sydnones with heterocyclic strained cycloalkynes.

The in situ generation of arynes or six-membered cycloalkynes from their corresponding trimethylsilyl triflates was recently used by Garg et al. [115] and Bräse et al. [116] in expanding the utility of oxygen- or nitrogen-containing strained heterocycloalkynes (Scheme 15) but the regioselectivity was poor in most cases.

### Photochemical reaction of sydnones with non-symmetrical alkynes

As mentioned in the previous section, Gotthardt and Reiter [63–64] studied the photochemical reaction of sydnones with terminal alkynes. They have also studied the reaction with phenylacetylene, methyl propiolate and ethyl phenylpropiolate in a batch reactor under irradiation with 300 nm light (Table 6).

**Table 6:** Photochemical cycloaddition of *N*-phenylsydnones with non-symmetrical alkynes.

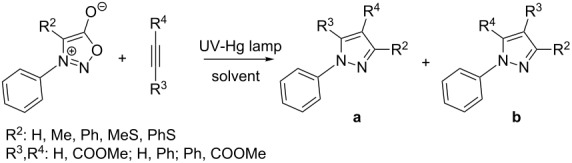							

entry	R^2^	R^3^	R^4^	conditions	ratio **a**:**b**	yield **a**/**b** [%]	ref.

1	Ph	H	COOMe	benzene, 23 h, light (300 nm)	16:84	12/62	[63–64]
2	Ph	COOEt	Ph	CH_2_Cl_2_, 27.5 h, light (300 nm)	0:100	0/33	[63–64]
3	Ph	H	Ph	CH_2_Cl_2_, 66 h, light (300 nm)	0:100	0/63	[63–64]
4	Me	H	Ph	CH_2_Cl_2_, 62 h, light (300 nm)	0:100	0/13	[64]
5	MeS	H	COOMe	CH_2_Cl_2_, 27 h, light (300 nm)	12:88	6/44	[64]
6	PhS	H	COOMe	benzene, 18.5 h, light (300 nm)	n.d.	5/41	[64]

The formation of both regioisomers **a** and **b** was observed when the most reactive methyl propiolate was used as a reactant. Moreover, the ratio (16:84) obtained from starting 3,4-diphenylsydnone is similar with those obtained from 1,3-diphenylnitrilimine independently generated either from 2,5-diphenyltetrazol or from *N*-phenylbenzenecarbohydrazonoyl chloride. This observation clearly supports the mechanism depicted in Scheme 6. The distribution of both regioisomers qualitatively agrees with the proposal of Houk et al. [94] combining the dipole HOMO with the dipolarophile LUMO (type-I mechanism).

### Copper-catalyzed reaction of sydnones with terminal alkynes

A substantial breakthrough in the field of 3-arylsydnone-terminal alkyne cycloaddition was achieved by Taran’s group in 2013 [3]. They developed a regioselective Cu(I)-phenanthroline-catalyzed variant of this reaction (i.e., copper-catalyzed sydnone alkyne cycloaddition; henceforth called CuSAC) enabling regioselective formation of 1,4-disubstituted pyrazoles under much milder reaction conditions (in various solvents including aqueous solution at 25–60 °C, Table 7) than previously used for its thermal-mediated counterpart. Such mild reaction conditions together with very high and reverse regioselectivity and efficiency (in most cases 85–99% yields) makes the CuSAC reaction a very good alternative to the well-established azide–alkyne click-reaction [117] useful not only in classical organic synthesis but also in bioconjugation applications. Moreover, a further improvement was later devised by the same authors, which avoids the highly toxic *N*-nitroso-*N*-phenylglycine, (precursor of sydnone) and involves a three-step one-pot transformation of starting *N*-phenylglycine to the corresponding pyrazole [118].

**Table 7:** Cu(I)-catalyzed cycloaddition of sydnones with terminal alkynes.

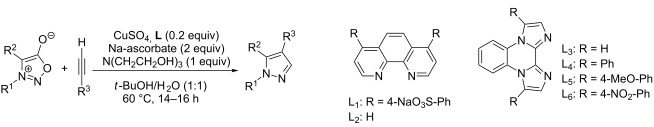						

entry	R^1^	R^2^	R^3^	ligand (L)	yield [%]	ref.

1	Ph	H	PhCH_2_CH_2_	L_1_ L_1_ L_2_	96–98 85^a^ 99	[3,119–120] [118] [3]
2	Ph	H	Ph	L_1_	80	[3,120]
3	Ph	H	4-MeOPh	L_1_	64	[3,120]
4	Ph	H	2-Py	L_1_	95 69^a^	[3] [118]
5	Ph	H	thiophen-3-yl	L_1_	95	[3,120]
6	Ph	H	1-heptyl	L_1_	61	[3,120]
7	Ph	H	PhCOOCH_2_	L_1_	93	[3,120]
8	Ph	H	(CH_3_)_2_C(OH)	L_1_	83	[3,120]
9	Ph	H	COOEt	L_1_	95 51^a^	[3,120] [118]
10	4-COOH-Ph	H	PhCH_2_CH_2_	L_1_	99	[3,120]
11	4-MeCO-Ph	H	PhCH_2_CH_2_	L_1_	97	[3]
12	4-COOH-Ph	H	(CH_3_)_2_C(OH)	L_1_	93	[3,120]
13	4-COOH-Ph	H	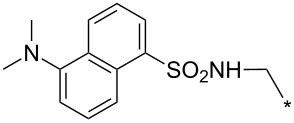	L_1_	99	[3,120]
14	Ph	H	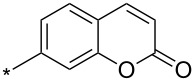	L_1_	85	[3]
15	Ph	H	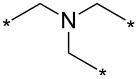	L_1_	85	[3,120]
16	Ph	H	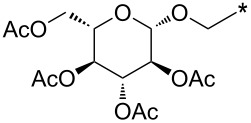	L_1_	96	[3,120]
17	Ph	H	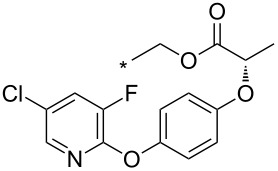	L_1_	62 55^a^	[3,120] [118]
18	Ph	H	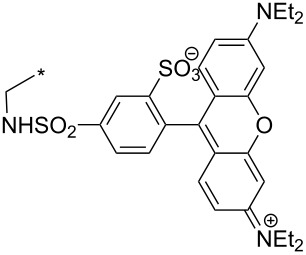	L_1_	92	[3,120]
19	Ph	H	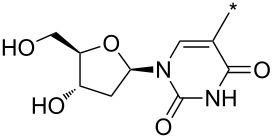	L_1_	84	[3,120]
20	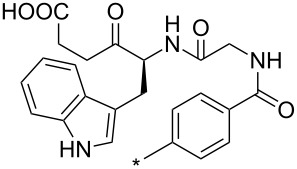	H	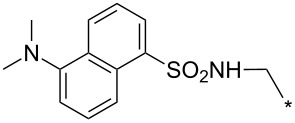	L_1_	99	[3,120]
21	Ph	H	Bn–N–Ts	L_2_	64	[116]
22	4-F-Ph	H	Bn–N–Ts	L_2_	54	[116]
23	4-CF_3_-Ph	H	Bn–N–Ts	L_2_	57	[116]
24	4-MeO-Ph	H	Bn–N–Ts	L_2_	57	[116]
25	4-MeO-Ph	H	PhCH_2_CH_2_	L_1_	69^a^	[118]
26	4-Me-Ph	H	PhCH_2_CH_2_	L_1_	72^a^	[118]
27	4-I-Ph	H	PhCH_2_CH_2_	L_1_	78^a^	[118]
28	4-NO_2_-Ph	H	PhCH_2_CH_2_	L_1_	69^a^	[118]
29	4-CN-Ph	H	PhCH_2_CH_2_	L_1_	92^a^	[118]
30	4-COOH-Ph	H	PhCH_2_CH_2_	L_1_	85^a^	[118]
31	4-CF_3_-Ph	H	PhCH_2_CH_2_	L_1_	80^a^	[118]
32	3-I-Ph	H	PhCH_2_CH_2_	L_1_	83^a^	[118]
33	naphthalen-1-yl	H	PhCH_2_CH_2_	L_1_	69^a^	[118]
34	2-COOMe-thiophen-3-yl	H	PhCH_2_CH_2_	L_1_	50^a^	[118]
35	Ph	H	Ph	L_1_	84^a^	[118]
36	Ph	H	*n*-pentyl	L_1_	82^a^	[118]
37	Ph	H	CH_2_NHCOO-*t*-Bu	L_1_	85^a^	[118]
38	Ph	H	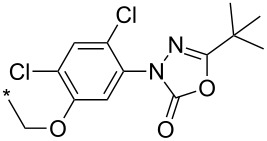	L_1_	91^a^	[118]
39	Ph	Br	PhCH_2_CH_2_	L_1_ L_2_ L_3_ L_4_ L_5_ L_6_	74^b^ 67^c^ 60^d^ 75 74 13	[119–120] [119] [119] [119] [119] [119]
40	4-Me-Ph	Br	PhCH_2_CH_2_	L_4_	80	[119]
41	4-MeO-Ph	Br	PhCH_2_CH_2_	L_4_	70	[119–120]
42	4-F-Ph	Br	PhCH_2_CH_2_	L_4_	55	[119]
43	4-I-Ph	Br	PhCH_2_CH_2_	L_4_	72	[119]
44	Ph	Br	COOEt	L_4_	38	[119]
45	Ph	Br	Ph	L_4_	63	[119]
46	Ph	Br	6-MeO-naphthalen-2-yl	L_4_	77	[119]
47	Ph	Br	4-MeO-Ph	L_4_	44	[119]
48	Ph	Br	CH_2_NHCOO-*t*-Bu	L_4_	69	[119]
49	Ph	Br	CH_2_OCOPh	L_4_	52	[119]
50	Ph	Br	BrCH_2_CH_2_	L_1_ L_4_	45^a^ 63	[119]
51	quinolin-5-yl	Br	PhCH_2_CH_2_	L_4_	33	[119]
52	Ph	Br	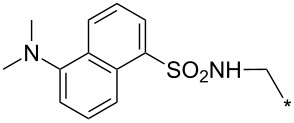	L_4_	52	[119]
53	Ph	Br	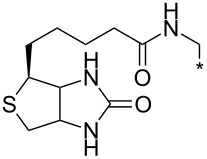	L_4_	65	[119]
54	Ph	Me	PhCH_2_CH_2_	L_1_	7	[119]
55	Ph	Cl	PhCH_2_CH_2_	L_1_	80^e^	[119]
56	Ph	CN	PhCH_2_CH_2_	L_1_	10^f^	[119]

^a^One-pot protocol starting from corresponding *N*-phenyl glycine; ^b^ratio 1,4,5:1,3,5 is 83:17; ^c^ratio 1,4,5:1,3,5:1,4,5-debrominated product is 83:10:7; ^d^ratio 1,4,5:1,3,5:1,4,5-debrominated product is 97:0:3; ^e^ratio 1,4,5:1,3,5 is 96:4; ^f^ratio 1,4,5:1,3,5 is 50:50.

**Scheme 16:**
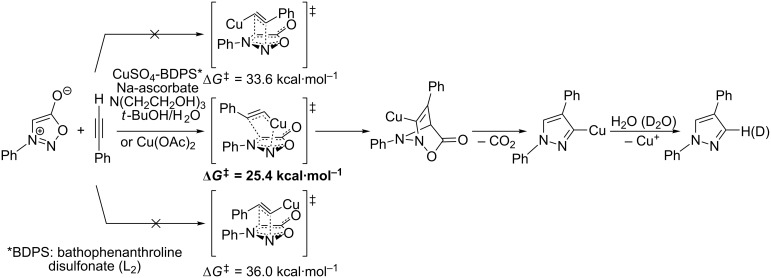
Mono-copper catalyzed cycloaddition reaction.

There are several limitations of the CuSAC reaction. First, it apparently fails with 3-alkyl sydnones and also with almost all 4-substituted 3-phenylsydnones except 4-F [44], 4-Cl and 4-Br derivatives [119]. However, this fortunate exception gave the further possibility to exchange halogen (especially bromine) by either an aryl, alkyl or alkenyl group via Suzuki coupling reaction with boronic acids to give otherwise rarely available 1,4,5-trisubstituted pyrazoles [119]. The second limitation is that the CuSAC reaction proceeds only with terminal alkynes. The latter fact clearly indicates some kind of participation of the alkyne’s slightly acidic terminal hydrogen in the reaction mechanism. Indeed, as early as in his primary paper [3] Taran suggested formation of Cu(I) acetylide (for additional information concerning reactions involving Cu(I) acetylides see references [121–122]) as the key species coordinating N2 of the sydnone through the Cu atom in the transition state. This suggestion was supported by Gomez-Bengoa and Harrity et al. [92] who performed thorough quantum calculation of various transition states involving different modes of interaction between 3-phenylsydnone and Cu(I) phenylacetylide (Scheme 16) and found Taran’s suggestion as the most plausible because of the lowest activation free energy (Δ*G*^‡^ = 25.4 kcal·mol^−1^) and due to the observed 1,4-regiocontrol. Intrinsic reaction coordinate (IRC) calculations also showed concerted but asynchronous formation of the pyrazole ring, through initial C–C bond formation followed by Cu–N dissociation and C–N bond formation. Experiments performed in *t*-BuOD/D_2_O [119] also showed almost exclusive (>98:2) deuteration of position 3 in the final pyrazole ring. This finding supports the idea of Cu(I)-acetylide addition to give 3-metalated pyrazole (Cu-pyrazolide) that is, in deuteric solvent hydrolyzed to give 3-deutero pyrazole.

**Scheme 17:**
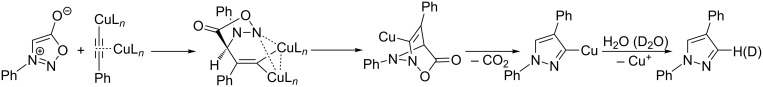
Di-copper catalyzed cycloaddition reaction.

However, Fokin et al. has recently [123] revealed that monomeric copper acetylide complexes are not reactive toward organic azides in analogous copper-catalyzed alkyne–azide cycloaddition (CuAAC) and the catalysis by an external Cu(I) salt is necessary. This means that a dinuclear copper complex – most probably copper(I) acetylide bearing the π-bound copper salt – plays a key role during the cycloaddition step. On the basis of a crossover experiment with an isotopically enriched ^63^Cu(I) salt it was concluded that the CuAAC involves addition of azide nitrogen N3 to π-bound copper of dinuclear copper complex with concerted addition of alkyne β-carbon to azide terminal nitrogen N1. An intermediate formed in which N3 is coordinated to both copper atoms undergoes fast ligand exchange between both copper atoms which makes them equivalent. Then the same N3 coordinating both Cu atoms attacks the terminal carbon of the polarized double bond with concerted cleavage of one of the two copper atoms. The copper triazolide formed in this way is then hydrolyzed to the final triazole. The same presumption (Scheme 17) concerning the role of the two Cu atoms was also adopted by Taran in his newer paper [119] but no experimental evidence for this mechanism has been given yet.

From previous studies it is known that for the spherically symmetric d^10^ Cu(I) ion, the common geometries are two-coordinate linear, three-coordinate trigonal planar, and four-coordinate tetrahedral [124]. Phenanthrolines form with Cu(I) at 1:1 ratio three-coordinated trigonal planar complexes or at 2:1 ratio tetra-coordinated tetrahedral complexes [125]. If Cu_2_(CN)_2_ (in which CN is isoelectronic with acetylide) is employed as a Cu(I) source then three-coordinated trigonal planar polymeric arrangement was observed [126]. From this observations it appears that mono- or dimeric three-coordinated trigonal planar Cu(I)-acetylide-phenanthroline complex should be the reactive species during CuSAC. This idea was supported by the fact that during the reaction of 4-bromosydnones with 4-phenylbut-1-yne [119] tridentate tris(benzimidazole) ligands completely failed and tris(triazole) ligands gave only poor to moderate yields (16–65%) even at 100 °C, whereas all the bidentate ligands (phenanthrolines L_1_, L_2_ and diimidazo[1,2-*a*:2',1'-*c*]quinoxalines L_3_–L_6_) were found to be more efficient both in terms of the isolated yield as well as the regioselectivity (see entry 39 in Table 7). From the comparison of phenanthroline (L_1_, L_2_) and diimidazo[1,2-*a*:2',1'-*c*]quinoxaline (L_3_–L_6_) complexes it appears that the higher angle between the two coordinative nitrogen atoms may have a positive impact on the catalytic efficiency.

Gomez-Bengoa and Harrity et al. [92] also inspected the role of Cu(I)/Cu(II) salts as well as other Lewis acids which could strengthen the electrophilicity of the starting sydnone under thermal reaction conditions. They found two competitive catalytic routes leading to different cycloaddition products. According to their original presumption some Lewis acids (TMSOTf < Zn(OAc)_2_ < MgBr_2_ < Cu(OTf)_2_) catalyzed the thermal reaction of phenylsydnone with phenylacetylene to give the expected 1,3-diphenylpyrazole in a ratio >10:1 over the 1,4-diphenyl isomer. Quantum calculations and IR measurements performed for the most active Cu(OTf)_2_ have shown that this salt coordinates to the sydnone oxygen carrying a negative charge which leads to an energy decrease of the sydnone LUMO and an increase of its electrophilicity. Also computed activation free energy (Δ*G*^‡^ = 25.4 kcal·mol^−1^) for the rate-limitting [3 + 2]-cycloaddition step leading to the 1,3-isomer was substantially lower if compared to the uncatalyzed reaction pathway (Δ*G*^‡^ = 32.5 kcal·mol^−1^).

If other Cu(II) salts were used as a catalyst then the ratio of 1,3-/1,4-isomers gradually changed from 90:10 to 3:97 (Table 8).

**Table 8:** Influence of Cu(II) salt (1 equiv) on reaction of phenyl sydnone (1 equiv) with phenylacetylene (2 equiv).

copper salt^a^	conversion after 20 min. in *o*-DCB at 140 °C (%)	ratio 1,3-/1,4-pyrazole

Cu(OTf)_2_	100	90:10
Cu(TFA)_2_	75	80:20
Cu(BF_4_)_2_	44	64:36
CuCl_2_·4H_2_O	74	48:52
CuCO_3_	29	27:73
Cu(acac)_2_	19	20:80
CuBr_2_	91	19:81
Cu(OAc)_2_	39	17:83
Cu(2-Et-hexanoate)_2_	88	8:92
Cu(hfacac)_2_	13	3:97

^a^acac – acetylacetonate; hfacac – hexafluoroacetylacetonate; TFA - trifluoroacetate.

A completely different ratio of both isomers was observed when Cu(II) carboxylates and acetylacetonates were employed instead of Cu(OTf)_2_. This was explained by different operating mechanisms. While Cu(OTf)_2_, Cu(TFA)_2_ and Cu(BF_4_)_2_ behave mainly as Lewis acids, other Cu(II) salts/complexes preferentially oxidize one equivalent of phenylacetylene to give 1,4-diphenylbuta-1,3-diyne (isolated in 80% yield) and the evolved Cu(I) salt then forms Cu(I) acetylide with a second equivalent of phenylacetylene. Thus formed Cu(I) acetylide is then responsible for gradual increasing of 1,4-pyrazole occurrence. Quantum calculations [92] and IR measurements performed for Cu(OAc)_2_ also show that the Lewis acid character of this salt is less pronounced and formation of the 1,3-diphenylpyrazole necessitates a much higher activation free energy (Δ*G*^‡^ = 41.4 kcal·mol^−1^) than for the uncatalyzed reaction. Formation of 1,4-diphenylpyrazole through Cu(I)-acetylide addition is then the clearly preferred reaction pathway. Moreover, Cu(OAc)_2_ acts as a very good catalyst not only in the reaction of parent phenylsydnone with phenylacetylene [92]. After appropriate prolongation of the reaction time it delivers the corresponding 1,4-disubstituted pyrazoles in good to excellent yields and with a regioselectivity ratio exceeding 95:5 (Table 9). It is worth noting that 3-benzyl sydnone (representative of otherwise unreactive 3-alkylsydnones) reacts with the highly reactive ethyl propiolate to give ethyl 1-benzylpyrazole-4-carboxylate in good yield.

**Table 9:** Cu(OAc)_2_-catalyzed cycloaddition.

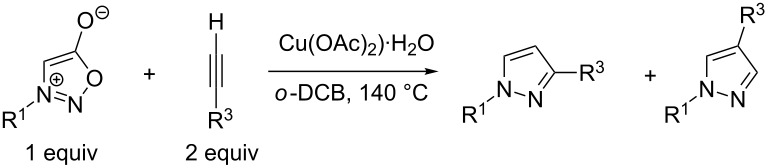					

entry	R^1^	R^3^	reaction time (h)	ratio 1,3:1,4	yield [%]

1	4-MeO-Ph	Ph	5	<5:95	53
2	Ph	COOEt	1	<5:95	93
3	4-MeO-Ph	COOEt	2	<5:95	81
4	Ph	*n*-Hex	3.5	<5:95	73
5	4-MeO-Ph	*n*-Hex	3.5	<5:95	54
6	Ph	cyclo-Hex	4	<5:95	100
7	4-MeO-Ph	cyclo-Hex	2.5	<5:95	71
8	Ph	cyclohex-1-enyl	2.5	<5:95	71
9	4-MeO-Ph	cyclohex-1-enyl	2.5	<5:95	60
10	Ph	cyclopropyl	4	<5:95	96
11	Ph	thiophen-3-yl	2.5	<5:95	95
12	4-F-Ph	COOEt	4	<5:95	95
13	Bn	COOEt	4	<5:95	60

Copper(II) acetate anchored on a modified silica gel can also serve as an efficient catalyst in batch reactor or if housed in stainless steel cartridges [127] in continuous-flow conditions (Table 10). Again, the 4-substituted pyrazole is preferentially formed.

**Table 10:** The solid-supported CuSAC reaction in batch or flow reactor.

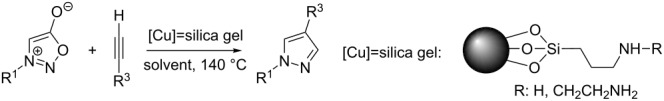					

entry	R^1^	R^3^	solvent	reaction (residence) time	yield [%]

1	Ph	Ph	*o*-DCB toluene	2 h (5 min)	100 100
2	Ph	COOEt	*o*-DCB toluene	6 h (5 min)	71 95
3	Ph	cyclopentyl	*o*-DCB toluene	6 h (15 min)	33 73
4	Ph	CH_2_OH	*o*-DCB toluene	20 h (15 min)	69 18
5	Ph	2-Py	*o*-DCB toluene	7 h (5 min)	70 24
6	4-MeO-Ph	Ph	*o*-DCB toluene	5 h (5 min)	85 75
7	4-MeO-Ph	COOEt	*o*-DCB toluene	6 h (5 min)	47 56
8	4-MeO-Ph	cyclopentyl	*o*-DCB toluene	6 h (15 min)	28 24
9	4-MeO-Ph	CH_2_OH	*o*-DCB toluene	20 h (15 min)	55 33
10	4-MeO-Ph	2-Py	*o*-DCB toluene	7.5 h (10 min)	68 26
11	Bn	Ph	*o*-DCB toluene	16 h (5 min)	47 77
12	Bn	COOEt	*o*-DCB	16 h	21

## Conclusion

Since its discovery in the sixties of the last century, the thermal [3 + 2]-cycloaddition of sydnones with alkynes represents a valuable synthetic tool for the preparation of polysubstituted pyrazoles and indazoles despite the limitations: the need for high temperatures (90–170 °C) and sometimes poorer regioselectivity. These obstacles can be surpassed either by suitable substitution (activating electron-withdrawing groups, removable silyl or carboxylate groups or replaceable boronic esters) or by efficient catalysis using Lewis acids. Preferential formation of 1,3-di- or 1,3,5-trisubstituted pyrazoles (>90:10) is observed in most cases when a terminal alkyne was used as a reactant. On the other hand, the recent discovery of Cu(I) catalysis in the sydnone–alkyne cycloaddition (CuSAC) enables regioselective formation of complementary 1,4-disubstituted or 5-halogeno-1,4-disubstituted pyrazoles under very mild reaction conditions (aqueous *t*-BuOH solution at 60 °C) and can be considered as a good illustration of the click-reaction. Another important example of sydnone cycloaddition involves a very fast reaction with strained seven- or eight-membered cycloalkynes (strain-promoted sydnone alkyne cycloaddition; SPSAC) which takes place without any catalyst and at ambient temperature. Such mild reaction conditions, (ultra) fast and unambiguous product formation make SPSAC useful in bio-orthogonal applications and competitive in comparison with analogous strain-promoted azide–alkyne cycloaddition (SPAAC). The last possibility of how to influence the cycloaddition between sydnones and alkynes involves photochemical performance of this reaction. Under UV-irradiation sydnones form the corresponding unstable nitrilimines which then undergo [3 + 2]-cycloaddition to give pyrazoles carrying substituents originating from alkynes in positions 4 and 5 instead of 3 and 4. Yields of this photochemical reaction are mostly lower than 50% which makes this method less convenient.
